# Supplementary material for: Progressive axonopathy when oligodendrocytes lack the myelin protein CMTM5
Source: eLife. 2022 Mar 11;11:e75523. doi: 10.7554/eLife.75523 (PMC8916772; doi:10.7554/eLife.75523)
Supplement: Figure 5—source code 1. [file elife-75523-fig5-code1.zip › Buscham Figure 5 Source code file 1.docx]

**Figure 5 Source code file 1.** Code used for analysis of VEP and ERG in Figure 5.

%%% ERGAnalysisfromBioSig_CMTM5.m

% analysis of ERG and VEP traces generated using Tucker Davis Technologies BioSig RP software

% used with Matlab 2016

% Written by Gerhard Hoch with modifications by Nicola Strenzke, both Institute for Auditory Neuroscience Göttingen, Germany

% contacts: ghoch@gwdg.de Nicola.Strenzke@med.uni.goettingen.de

% current version February 04th, 2022

%

% The routine will read the *.arf files indicated in mice(x).path

% (we used 19531 data points for an acquisition duration of 799.9 ms,

% stimulus onset was at 50ms, the Attenuator was used to adjust light

% intensity. In the original version, filename elements phot/scot indicated

% standardized recording schemes under photopic and scotopic conditions

% which corresponded to calibration values entered into the standardized

% excel tables. This naming was not used in the CMTM5 project.

% The routine will then filter the traces, find the A and B waves

% and enter the resulting data (amplitudes, latencies of A and B waves, amplitude of oscillatory potentials)

% in the corresponding sheets and rows/columns of a standardized Excel file.

% XLSPosA=['AK'];mice(1).XLSPosB=['BA'];mice(1).XLSPosO=['D'];mice(1).XLSPosOFreq=['U'] indicate the columns

% mice(1).ERGAnalysisXLS='W:\public\Data\CMTM5_VEP\21-09-09_ERG_Analysis_CMTM5' indicates the name of the Excel file.

%

%

% For VEP analysis in CMTM5 manuscript we used the following settings

% FcAWave=400; %low pass corner frequency for A wave, adapted for VEP

% FcBWave=400; % low pass corner frequency for B wave, adapted for VEP

% and later

% MaxTimeFFTInd=uint32(Fs*0.2);

% MaxTimeAmakrin=uint32(Fs*0.2);

% MaxTimeBWave=uint32(Fs*0.18);% defines where B Wave is looked for (starting from stimulustime). A wave is between stimulus and B wave.

% and inverted the original traces.

clear all;

mice(1).path= ['C:\Users\nstrenz\Documents\data\VEP-ERG_CMTM5\KO-ERG\ERGscot1_24_1'];mice(1).NrGroup=0;mice(1).XLSPosA=['AK'];mice(1).XLSPosB=['BA'];mice(1).XLSPosO=['D'];mice(1).XLSPosOFreq=['U'];mice(1).ERGAnalysisXLS='W:\public\Data\CMTM5_VEP\21-09-09_ERG_Analysis_CMTM5';

mice(2).path= ['C:\Users\nstrenz\Documents\data\VEP-ERG_CMTM5\KO-ERG\ERCscot1_25_1'];mice(2).NrGroup=0;mice(2).XLSPosA=['AL'];mice(2).XLSPosB=['BB'];mice(2).XLSPosO=['E'];mice(2).XLSPosOFreq=['V'];mice(2).ERGAnalysisXLS='W:\public\Data\CMTM5_VEP\21-09-09_ERG_Analysis_CMTM5';

% add additional datasets here as needed

nmice=size(mice,2);

doXLSERGAnalysis=1; % set to 1 if you want the standardized excel table analysis

doSumPlot=1; % set to 1 if you want a jpg file with the result overview.

doXLSArray=0; % set to 1 if you want the XLS array (not recently tested)

doXLSAccess=0; % set to 1 if you want our data in MS access format (not recently tested)

doAllPlot=0; % set to 1 if you want plots of each recording

NoiseSchwelle=0.000020; % noise threshold

%NoiseSchwelle=0.000010;

StimulusTime=50; % delay of stimulus in ms compared to recording onset

FcAWave=400; %low pass corner frequency for A wave

FcBWave=20; % low pass corner frequency for B wave

GesamtFiles=1;

lAccess=1;

for j=1:size(mice,2)

if size(mice(j).path,2)>0

%close all;

StimulusDurPhot=0;

StimulusDurScot=0;

MaxMeanBSAllScopReccounter=1;

MaxMeanBSAllPhotReccounter=1;

ispath=regexprep(mice(j).path, ' ','')

s0=strfind(ispath,'\');s0Size=size(s0,2);

isLastDir= ispath(s0(s0Size-1)+1:s0(s0Size)-1);isLastDir=regexprep(isLastDir,'_',' ');

isGenoTyp= ispath(s0(s0Size-2)+1:s0(s0Size-1)-1);

isGenoTypDir=ispath(1:s0(s0Size-1));

%if isdir(ispath)

%files=dir(fullfile(ispath,'*1.arf'));

files=mice(j).path %adjusted 2021 to directly enter filenames above.

nFiles=size(files);

if nFiles(1:1)>0

%if doSumPlot==1 mkdir(fullfile(ispath,'sum\')); end;

if doSumPlot==1 mkdir(ispath,'sum\'); end;

if doXLSERGAnalysis==1

end;

%for i=1:nFiles(1:1)

for i=1:1

clear MaxMeanBSAllPhot DiffAmpAmakrinPhot IndexBFiltPhot DiffAmpBFiltPhot IndexAPhot DiffAmpAFirstMinPhot CandelaPhot

clear MaxMeanBSAllScop DiffAmpAmakrinScot IndexBFiltScot DiffAmpBFiltScot IndexAScot DiffAmpAFirstMinScot CandelaScot

clear Candela Amakrin AmakrinFilt DiffAmpAmakrin MaxMeanBSorg MaxMeanBSBWaveFilt BSFilt DiffAmpAmakrinFreq

reccounter=0;

ModeGenType=0;

ModeStimTime=0;

StimTime=0.0001;

%[pathstr,name,ext]=fileparts(files(i).name);

[pathstr,name,ext]=fileparts(files);

ext='.arf'

%name=ispath

[num2str(j) '(' num2str(nmice) ') ' num2str(i) '(' num2str(nFiles(1:1)) ')' num2str(GesamtFiles) ' ' name]

if strfind(isLastDir,'WT')>0 ModeGenType=1; end;

if strfind(isLastDir,'KO')>0 ModeGenType=2; end;

%if ((strfind(name,'phot')>0) | (strfind(name,'scot')>0))

%else name=[name ' scot'];end;

if (strfind(name,'phot')>0)

StimulusDurPhot=StimulusDurPhot+1;

if strfind(name,'1')>0 StimTime=0.0001; ModeStimTime=1; end

if strfind(name,'2')>0 StimTime=0.0001; ModeStimTime=2; end

if strfind(name,'3')>0 StimTime=0.001; ModeStimTime=3; end

if strfind(name,'4')>0 StimTime=0.005; ModeStimTime=4; end

end

if (strfind(name,'scot')>0)

StimulusDurScot=StimulusDurScot+1;

if strfind(name,'1')>0 StimTime=0.0001; ModeStimTime=5; end

if strfind(name,'2')>0 StimTime=0.001; ModeStimTime=6; end

if strfind(name,'3')>0 StimTime=0.005; ModeStimTime=7; end

end

StimTime=0.0001; ModeStimTime=5; % Nicola 2021, I only used Scot1

clear RecHeadType GroupType RecType

l=1;

%fid=fopen(ispath,files(i).name),'r');

fid=fopen([ispath,'.arf'],'r');

if fid==-1; message='file read error'; end

if fid>-1

RecHeadType.ftype = fread(fid,1,'int16');

RecHeadType.ngrps = fread(fid,1,'int16');

RecHeadType.nrecs = fread(fid,1,'int16');

RecHeadType.grpseek = fread(fid,200,'int32');

RecHeadType.recseek = fread(fid,2000,'int32');

RecHeadType.fhand = fread(fid,1,'int32');

if (mice(j).NrGroup==0) doNrGroup=RecHeadType.ngrps;

else doNrGroup=mice(j).NrGroup;

end;

for group=1:doNrGroup

GroupType(group).grpn = fread(fid,1,'int16');

GroupType(group).frecn = fread(fid,1,'int16');

GroupType(group).nrecs = fread(fid,1,'int16');

GroupType(group).SI.id = char(fread(fid,16,'char'));

GroupType(group).SI.ref(1,1:16) = char(fread(fid,16,'char'));

GroupType(group).SI.ref(2,1:16) = char(fread(fid,16,'char'));

GroupType(group).SI.memo = char(fread(fid,50,'char'));

GroupType(group).beg_t = (fread(fid,1,'int32'));

GroupType(group).end_t = (fread(fid,1,'int32'));

GroupType(group).sgfname = char(fread(fid,200,'char'));

for x=1:10

GroupType(group).vname(x,1:15) = char(fread(fid,15,'char'));

end

for x=1:10

GroupType(group).units(x,1:5) = char(fread(fid,5,'char'));

end

GroupType(group).srate = fread(fid,1,'float32');

GroupType(group).cc_t = fread(fid,1,'int32');

GroupType(group).version = fread(fid,1,'int16');

GroupType(group).postproc = fread(fid,1,'int32');

GroupType(group).dump = fread(fid,92,'char');

for rec=1:GroupType(group).nrecs % LOOP ON RECORDS

clear yFFT FFTSize

RecType(group,rec).recn = fread(fid,1,'int16');

RecType(group,rec).grpid = fread(fid,1,'int16');

RecType(group,rec).grp_t = fread(fid,1,'int32');

RecType(group,rec).newgrp = fread(fid,1,'int16');

RecType(group,rec).sgi = fread(fid,1,'int16');

RecType(group,rec).chan = (fread(fid,1,'int8'));

RecType(group,rec).rtype = char(fread(fid,1,'char'));

RecType(group,rec).npts = fread(fid,1,'int16');

RecType(group,rec).osdel = fread(fid,1,'float32');

RecType(group,rec).dur = fread(fid,1,'float32');

RecType(group,rec).srate = fread(fid,1,'float32');

RecType(group,rec).arthresh = fread(fid,1,'float32');

RecType(group,rec).gain = fread(fid,1,'float32');

RecType(group,rec).accouple = fread(fid,1,'int16');

RecType(group,rec).navgs = fread(fid,1,'int16');

RecType(group,rec).narts = fread(fid,1,'int16');

RecType(group,rec).beg_t1 = fread(fid,1,'uint8');

RecType(group,rec).beg_t2 = fread(fid,1,'uint8');

RecType(group,rec).beg_t3 = fread(fid,1,'uint8');

RecType(group,rec).beg_t4 = fread(fid,1,'uint8');

RecType(group,rec).end_t1 = fread(fid,1,'uint8');

RecType(group,rec).end_t2 = fread(fid,1,'uint8');

RecType(group,rec).end_t3 = fread(fid,1,'uint8');

RecType(group,rec).end_t4 = fread(fid,1,'uint8');

RecType(group,rec).vv = fread(fid,10,'float32');

RecType(group,rec).CUR = fread(fid,360,'char'); %just read in the cursor info to keep alignment, not going to use

GesamtFiles=GesamtFiles+1; reccounter=reccounter+1;

if (reccounter==1) clear Pyy PyyFilt CalcData FiltMaxMeanBS MaxMeanBS Candela DiffAmpA DiffAmpB DiffAmpAFilt DiffAmpBFilt DiffAmpAFirstMin IndexA IndexB IndexAFilt IndexBFilt IndexAFirstMin DiffAmpAmakrin

end

BS(reccounter).vv=RecType(group,rec).vv;

BS(reccounter).vname=GroupType(group).vname;

Pt=RecType(group,rec).npts;

BS(reccounter).data = fread(fid,Pt,'float32');

CalcData=BS(reccounter).data;

BS(reccounter).navgs=RecType(group,rec).navgs;

BS(reccounter).id=GroupType(group).SI.id;

BS(reccounter).ref(1,:)=GroupType(group).SI.ref(1,1:16);

BS(reccounter).ref(2,:)=GroupType(group).SI.ref(2,1:16);

BS(reccounter).srate=RecType(group,rec).srate;

BS(reccounter).chan=RecType(group,rec).chan;

t=1:RecType(group,rec).npts; t=t*RecType(group,rec).srate*0.001; t=t';

idS=sscanf(BS(reccounter).id,'%s');idS=regexprep(idS,'\','-');

refS=sscanf(BS(reccounter).ref(1,:),'%s');

SaveName=[isLastDir,' ',name,' ',num2str(BS(reccounter).vv(1,:)*10,'%3.0f'),' ',num2str(group),' ',num2str(rec)];

att=BS(reccounter).vv(1,:);

Fs=1000000/(RecType(group,rec).srate);

MeanMaxInd=uint32(Fs*0.040);

StimStartInd=uint32(Fs*StimulusTime/1000);

StimEndInd=uint32(Fs*StimulusTime/1000+0.008);

MaxTimeFFTInd=uint32(Fs*0.130);

MaxTimeAmakrin=uint32(Fs*0.130);

MaxTimeBWave=uint32(Fs*0.130);% defines where B Wave is looked for (starting from stimulustime). A wave is between stimulus and B wave.

PlotTimePt=RecType(group,rec).npts/2;

% filtering or A wave amplitude

N=2; [b,a]=butter(N, FcAWave/(Fs/2),'low');

BSFilt(reccounter,:)=filtfilt(b,a,BS(reccounter).data(:)); %filtered original trace

MaxMeanBSorg(reccounter,:)=BS(reccounter).data(:)-BSFilt(reccounter,StimEndInd);

MaxMeanBS(reccounter,:)=filtfilt(b,a,MaxMeanBSorg(reccounter,:));

% filtering for B wave ammplitude

N=2; [b,a]=butter(N, FcBWave/(Fs/2),'low');

MaxMeanBSBWaveFilt(reccounter,:)=filtfilt(b,a,MaxMeanBSorg(reccounter,:));

FilterPt=uint32(Fs/500);

FiltMaxMeanBS(reccounter,:)=MaxMeanBS(reccounter,:);

for iFilt=FilterPt+1:((RecType(group,rec).npts)-FilterPt)

FiltMaxMeanBS(reccounter,iFilt)=mean(MaxMeanBS(reccounter,iFilt-FilterPt:iFilt+FilterPt));

end;

[DiffAmpB(reccounter) IndexB(reccounter)]=max(MaxMeanBS(reccounter,StimEndInd:StimEndInd+MaxTimeBWave));

[DiffAmpA(reccounter) IndexA(reccounter)]=min(MaxMeanBS(reccounter,StimEndInd:StimEndInd+IndexB(reccounter)));

DiffAmpBAmpA(reccounter)=DiffAmpB(reccounter)-DiffAmpA(reccounter);

[DiffAmpBFilt(reccounter) IndexBFilt(reccounter)]=max(MaxMeanBSBWaveFilt(reccounter,StimEndInd:StimEndInd+MaxTimeBWave));

[DiffAmpAFilt(reccounter) IndexAFilt(reccounter)]=min(FiltMaxMeanBS(reccounter,StimEndInd:StimEndInd+IndexB(reccounter)));

DiffAmpBAmpAFilt(reccounter)=DiffAmpBFilt(reccounter)-DiffAmpAFilt(reccounter);

%changed by Nicola: subtract trace offset determined in a window

%between beginning of trace and up to 10 poin before stimulus onset

[DiffAmpAFirstMinAbs(reccounter) IndexAFirstMin(reccounter)]=min(MaxMeanBS(reccounter,StimEndInd:StimEndInd+abs(IndexAFilt(reccounter)-FilterPt)));

DiffAmpAFirstMin(reccounter)=DiffAmpAFirstMinAbs(reccounter)-mean(MaxMeanBS(reccounter,10:StimEndInd-50));

%Candela(reccounter)=((2.8553*att*att)-(178.25*att)+2779.4)*StimTime;

Candela(reccounter)=((2.4471*att*att)-(156.04*att) + 2477.5)*StimTime;% ab 17052013

N=2; Fc=30; [b,a] = butter(N, Fc/(Fs/2), 'high'); % high pass filter for oscillatory potentials/amakrin cell response

Amakrin(reccounter,:)=MaxMeanBS(reccounter,StimEndInd:MaxTimeAmakrin);

AmakrinFilt(reccounter,:)=filter(b,a,Amakrin(reccounter,:)); AmakrinFiltSize=size(AmakrinFilt(reccounter,:));

[DiffAmpAmakrinMax DiffAmpAmakrinMaxInd]=(max(AmakrinFilt(reccounter,:)));

DiffAmpAmakrin(reccounter)=(max(AmakrinFilt(reccounter,:)))-(min(AmakrinFilt(reccounter,:)));

if abs(DiffAmpAmakrin(reccounter))>NoiseSchwelle

isInd=uint32(DiffAmpAmakrinMaxInd+1);isDiffAmpAmakrinLast=DiffAmpAmakrinMax;isAmakrinFiltSize=uint32(AmakrinFiltSize(1,2)-1);isEnde=0;

if (isInd<isAmakrinFiltSize)

while ((isEnde==0) & (AmakrinFilt(reccounter,isInd)<isDiffAmpAmakrinLast))

isDiffAmpAmakrinLast=AmakrinFilt(reccounter,isInd);

if (isInd<isAmakrinFiltSize) isInd=isInd+1;

else isEnde=1; end;

end

end;

if (isInd+1<AmakrinFiltSize(1,2))

DiffAmpAmakrinMinInd=isInd; DiffAmpAmakrinFreq(reccounter)=Fs/(abs(DiffAmpAmakrinMinInd-DiffAmpAmakrinMaxInd)*2);

else

DiffAmpAmakrinFreq(reccounter)=NaN;

end;

else

DiffAmpAmakrinFreq(reccounter)=NaN;

end;

if strfind(name,'phot')>0

MaxMeanBSAllPhot(MaxMeanBSAllPhotReccounter,:)=MaxMeanBS(reccounter,:);MaxMeanBSAllPhotReccounter=MaxMeanBSAllPhotReccounter+1;

DiffAmpAmakrinPhot(reccounter,StimulusDurPhot)=DiffAmpAmakrin(reccounter);

IndexBFiltPhot(reccounter,StimulusDurPhot)=IndexBFilt(reccounter);

DiffAmpBFiltPhot(reccounter,StimulusDurPhot)=DiffAmpBFilt(reccounter);

IndexAPhot(reccounter,StimulusDurPhot)=IndexA(reccounter);

DiffAmpAFirstMinPhot(reccounter,StimulusDurPhot)=DiffAmpAFirstMin(reccounter);

CandelaPhot(reccounter,StimulusDurPhot)=Candela(reccounter);

end

if strfind(name,'scot')>0

MaxMeanBSAllScop(MaxMeanBSAllScopReccounter,:)=MaxMeanBS(reccounter,:);MaxMeanBSAllScopReccounter=MaxMeanBSAllScopReccounter+1;

DiffAmpAmakrinScot(reccounter,StimulusDurScot)=DiffAmpAmakrin(reccounter);

IndexBFiltScot(reccounter,StimulusDurScot)=IndexBFilt(reccounter);

DiffAmpBFiltScot(reccounter,StimulusDurScot)=DiffAmpBFilt(reccounter);

IndexAScot(reccounter,StimulusDurScot)=IndexA(reccounter);

DiffAmpAFirstMinScot(reccounter,StimulusDurScot)=DiffAmpAFirstMin(reccounter);

CandelaScot(reccounter,StimulusDurScot)=Candela(reccounter);

end

if doXLSArray==1

if l==1

clear XLSArray

XLSArray(1,l)={['Att']};

XLSArray(2,l)={['Stimulus']};

XLSArray(3,l)={['Stimulus*Zeit']};

XLSArray(4,l)={['A-Wave +']};

XLSArray(5,l)={['A-Wave time +']};

XLSArray(6,l)={['B-Wave +']};

XLSArray(7,l)={['B-Wave time +']};

XLSArray(8,l)={['Filt A-Wave x']};

XLSArray(9,l)={['Filt A-Wave time x']};

XLSArray(10,l)={['Filt B-Wave x']};

XLSArray(11,l)={['Filt B-Wave time x']};

XLSArray(12,l)={['First A-Wave o']};

XLSArray(13,l)={['First A-Wave time o']};

XLSArray(14,l)={['DiffAmpOszillation']};

XLSArray(15,l)={['Att']};

for IsEXCEL=1:RecType(group,rec).npts

XLSArray(IsEXCEL+15,l)={t(IsEXCEL)};

end

end

l=l+1;

XLSArray(1,l)={num2str(BS(reccounter).vv(1,:))};

XLSArray(2,l)={((2.4471*att*att)-(156.04*att) + 2477.5)};

XLSArray(3,l)={((2.4471*att*att)-(156.04*att) + 2477.5)*StimTime};

XLSArray(4,l)={DiffAmpA(reccounter)};

if abs(DiffAmpA(reccounter))>NoiseSchwelle XLSArray(5,l)={t(IndexA(reccounter)+(StimEndInd-StimStartInd))}; end;

XLSArray(6,l)={DiffAmpBAmpA(reccounter)};

if abs(DiffAmpBAmpA(reccounter))>NoiseSchwelle XLSArray(7,l)={t(IndexB(reccounter)+(StimEndInd-StimStartInd))}; end;

XLSArray(8,l)={DiffAmpAFilt(reccounter)};

if abs(DiffAmpAFilt(reccounter))>NoiseSchwelle XLSArray(9,l)={t(IndexAFilt(reccounter)+(StimEndInd-StimStartInd))}; end;

XLSArray(10,l)={DiffAmpBAmpAFilt(reccounter)};

if abs(DiffAmpBAmpAFilt(reccounter))>NoiseSchwelle XLSArray(11,l)={t(IndexBFilt(reccounter)+(StimEndInd-StimStartInd))}; end;

XLSArray(12,l)={DiffAmpAFirstMin(reccounter)};

if abs(DiffAmpAFirstMin(reccounter))>NoiseSchwelle XLSArray(13,l)={t(IndexAFirstMin(reccounter)+(StimEndInd-StimStartInd))}; end;

XLSArray(14,l)={DiffAmpAmakrin(reccounter)};

XLSArray(15,l)={num2str(BS(reccounter).vv(1,:))};

for IsEXCEL=1:RecType(group,rec).npts

XLSArray(IsEXCEL+15,l)={BS(reccounter).data(IsEXCEL)};

end;

end;

if doXLSAccess==1

if lAccess==1

XLSArrayAccess={'fname','SName','LastDir','DateMeasure','Typ','Gen','Att','Stimulus','Stimulus*Zeit',...

'A-Wave +','A-Wave time +','B-Wave +','B-Wave time +',...

'Filt A-Wave x','Filt A-Wave time x','Filt B-Wave x','Filt B-Wave time x',...

'First A-Wave o','First A-Wave time o','DiffAmpOszillation',...

};end;

lAccess=lAccess+1;

XLSArrayAccess(lAccess,1)={files(i).name};

XLSArrayAccess(lAccess,2)={ispath};

XLSArrayAccess(lAccess,3)={isLastDir};

XLSArrayAccess(lAccess,4)={files(i).date};

if strfind(isLastDir,'Triple41')>0 Gen='Triple41';end;

if strfind(isLastDir,'NLG1')>0 Gen='NLG1';end;

if strfind(isLastDir,'NLG2')>0 Gen='NLG2';end;

if strfind(isLastDir,'NLG3')>0 Gen='NLG3';end;

if strfind(isLastDir,'NLG4')>0 Gen='NLG4';end;

if strfind(isLastDir,'STO2')>0 Gen='STO2';end;

if strfind(isLastDir,'CAST')>0 Gen='CAST';end;

if strfind(isLastDir,'C57N')>0 Gen='C57N';end;

if strfind(isLastDir,'Otof')>0 Gen='Otof';end;

if strfind(isLastDir,'CASTIIdhet')>0 Gen='CASTIIdhet';end;

XLSArrayAccess(lAccess,5)={Gen};

if strfind(isLastDir,'WT')>0 Typ='WT'; end;

if strfind(isLastDir,'KO')>0 Typ='KO'; end;

XLSArrayAccess(lAccess,6)={Typ};

XLSArrayAccess(lAccess,7)={num2str(BS(reccounter).vv(1,:))};

XLSArrayAccess(lAccess,8)={((2.4471*att*att)-(156.04*att) + 2477.5)};

XLSArrayAccess(lAccess,9)={((2.4471*att*att)-(156.04*att) + 2477.5)*StimTime};

XLSArrayAccess(lAccess,10)={DiffAmpA(reccounter)};

XLSArrayAccess(lAccess,11)={t(IndexA(reccounter)+(StimEndInd-StimStartInd))};

XLSArrayAccess(lAccess,12)={DiffAmpBAmpA(reccounter)};

XLSArrayAccess(lAccess,13)={t(IndexB(reccounter)+(StimEndInd-StimStartInd))};

XLSArrayAccess(lAccess,14)={DiffAmpAFilt(reccounter)};

XLSArrayAccess(lAccess,15)={t(IndexAFilt(reccounter)+(StimEndInd-StimStartInd))};

XLSArrayAccess(lAccess,16)={DiffAmpBAmpAFilt(reccounter)};

XLSArrayAccess(lAccess,17)={t(IndexBFilt(reccounter)+(StimEndInd-StimStartInd))};

XLSArrayAccess(lAccess,18)={DiffAmpAFirstMin(reccounter)};

XLSArrayAccess(lAccess,19)={t(IndexAFirstMin(reccounter)+(StimEndInd-StimStartInd))};

XLSArrayAccess(lAccess,20)={DiffAmpAmakrin(reccounter)};

end;

if doXLSERGAnalysis==1

if strfind(isLastDir,'WT')>0 XLSSheetScot='scot WT'; XLSSheetPhot='phot WT';XLSSheetScotLat='scot WT Lat'; XLSSheetPhotLat='phot WT Lat';XLSSheetScotOsc='scot WT Osc'; XLSSheetPhotOsc='phot WT Osc';end;

if strfind(isLastDir,'KO')>0 XLSSheetScot='scot KO'; XLSSheetPhot='phot KO';XLSSheetScotLat='scot KO Lat'; XLSSheetPhotLat='phot KO Lat';XLSSheetScotOsc='scot KO Osc'; XLSSheetPhotOsc='phot KO Osc';end;

if strfind(isLastDir,'HET')>0 XLSSheetScot='scot HET'; XLSSheetPhot='phot HET';XLSSheetScotLat='scot HET Lat'; XLSSheetPhotLat='phot HET Lat';XLSSheetScotOsc='scot HET Osc'; XLSSheetPhotOsc='phot HET Osc';end;

%if (strfind(name,'phot')>0) %taken out Nicola 04-21 for

%CMTM5

attPhot=round(double(BS(reccounter).vv(1,:)));

switch attPhot;

case 30

XLSline=15;

case 29

XLSline=16;

case 28

XLSline=17;

case 27

XLSline=18;

case 26

XLSline=19;

case 25

XLSline=20;

case 24

XLSline=21;

case 23

XLSline=22;

case 22

XLSline=23;

case 21

XLSline=24;

case 20

XLSline=25;

case 15

XLSline=26;

case 10

XLSline=27;

case 5

XLSline=28;

case 0

XLSline=29;

otherwise

XLSline=0

attPhot

end

if (XLSline>0 )

%if strfind(name,'3')>0 XLSline=XLSline+25; end;

%if strfind(name,'4')>0 XLSline=XLSline+50; end;

xlswrite(mice(j).ERGAnalysisXLS, DiffAmpBAmpAFilt(reccounter)*1000000, XLSSheetPhot, [mice(j).XLSPosB num2str(XLSline)]);

xlswrite(mice(j).ERGAnalysisXLS, DiffAmpAFirstMin(reccounter)*(-1)*1000000, XLSSheetPhot, [mice(j).XLSPosA num2str(XLSline)]);

if abs(DiffAmpBAmpAFilt(reccounter))>NoiseSchwelle

xlswrite(mice(j).ERGAnalysisXLS,t(IndexBFilt(reccounter)+(StimEndInd-StimStartInd)),XLSSheetPhotLat,[mice(j).XLSPosB num2str(XLSline)]);

end

if abs(DiffAmpAFirstMin(reccounter))>NoiseSchwelle

xlswrite(mice(j).ERGAnalysisXLS, t(IndexAFirstMin(reccounter)+(StimEndInd-StimStartInd)),XLSSheetPhotLat, [mice(j).XLSPosA num2str(XLSline)]);

end

xlswrite(mice(j).ERGAnalysisXLS,DiffAmpAmakrin(reccounter)*1000000, XLSSheetPhotOsc, [mice(j).XLSPosO num2str(XLSline)]);

if abs(DiffAmpAmakrin(reccounter))>NoiseSchwelle

xlswrite(mice(j).ERGAnalysisXLS,DiffAmpAmakrinFreq(reccounter), XLSSheetPhotOsc, [mice(j).XLSPosOFreq num2str(XLSline)]);

end;

end

end

if (strfind(name,'scot')>0)

attScot=uint32(double(BS(reccounter).vv(1,:)*10));

switch attScot

case 300

XLSline=15;

case 299

XLSline=16;

case 298

XLSline=17;

case 297

XLSline=18;

case 296

XLSline=19;

case 295

XLSline=20;

case 294

XLSline=21;

case 293

XLSline=22;

case 292

XLSline=23;

case 291

XLSline=24;

case 290

XLSline=25;

case 280

XLSline=26;

case 270

XLSline=27;

case 260

XLSline=28;

case 250

XLSline=29;

case 240

XLSline=30;

case 230

XLSline=31;

case 220

XLSline=32;

case 210

XLSline=33;

case 200

XLSline=34;

case 150

XLSline=35;

case 100

XLSline=36;

case 50

XLSline=37;

case 0

XLSline=38;

otherwise

XLSline=0

attScot

%end

if (XLSline>0 )

%if strfind(name,'2')>0 XLSline=XLSline+38; end;

%if strfind(name,'3')>0 XLSline=XLSline+74; end;

if (mice(j).XLSPosB)>0

xlswrite(mice(j).ERGAnalysisXLS, DiffAmpBAmpAFilt(reccounter)*1000000, XLSSheetScot, [mice(j).XLSPosB num2str(XLSline)]);

end;

if (mice(j).XLSPosA)>0

xlswrite(mice(j).ERGAnalysisXLS, DiffAmpAFirstMin(reccounter)*(-1)*1000000, XLSSheetScot, [mice(j).XLSPosA num2str(XLSline)]);

end;

if abs(DiffAmpBAmpAFilt(reccounter))>NoiseSchwelle

if (mice(j).XLSPosB)>0

xlswrite(mice(j).ERGAnalysisXLS, t(IndexBFilt(reccounter)+(StimEndInd-StimStartInd)), XLSSheetScotLat, [mice(j).XLSPosB num2str(XLSline)]);

end;

end;

if abs(DiffAmpAFirstMin(reccounter))>NoiseSchwelle

if (mice(j).XLSPosA)>0

xlswrite(mice(j).ERGAnalysisXLS, t(IndexAFirstMin(reccounter)+(StimEndInd-StimStartInd)), XLSSheetScotLat, [mice(j).XLSPosA num2str(XLSline)]);

end;

end

if (mice(j).XLSPosO)>0

xlswrite(mice(j).ERGAnalysisXLS,DiffAmpAmakrin(reccounter)*1000000, XLSSheetScotOsc, [mice(j).XLSPosO num2str(XLSline)]);

if abs(DiffAmpAmakrin(reccounter))>NoiseSchwelle

xlswrite(mice(j).ERGAnalysisXLS,DiffAmpAmakrinFreq(reccounter), XLSSheetScotOsc, [mice(j).XLSPosOFreq num2str(XLSline)]);

end;

end;

end

end

end;

end % record loop

end % group loop

fclose(fid);

end;

if doXLSArray==1

delete(fullfile(ispath,[name 'plot.xls'])); xlswrite(fullfile(ispath,[name 'plot']),XLSArray,'Sheet1');

end

if doSumPlot==1

h=figure;

subplot(2,4,1:2); plot(t(1:uint32(PlotTimePt)),MaxMeanBS(:,(1:uint32(PlotTimePt))));

set(gca,'FontSize',12);title(sprintf('%s',[' ' isLastDir ' ' name]),'FontSize',9);

xlabel(['time (msec) stimulus:' num2str(StimTime)],'FontSize',12);

ylabel('amplitude (V)','FontSize',12);

subplot(4,4,3); plot(t(StimEndInd:MaxTimeAmakrin),AmakrinFilt(:,:));

title(sprintf('%s',['oscillatory']),'FontSize',9);

xlabel(['time (msec)'],'FontSize',8); ylabel('amplitude (V)','FontSize',8);

% subplot(4,4,4);plot(tFFT(FFTIndMin:FFTIndMax),(Pyy(:,FFTIndMin:FFTIndMax)));

% xlabel(['frequenz (Hz)'],'FontSize',8); ylabel('amplitude (V)','FontSize',8);

subplot(4,4,4);plot(log10(Candela),DiffAmpAmakrinFreq,'ro');set(gca,'xlim',[-3 1]);

xlabel(['stimulus intensity (log cds/m²)'],'FontSize',8);ylabel('frequency (Hz)','FontSize',8);

subplot(4,2,4); plot(log10(Candela),DiffAmpAmakrin,'ro');

xlabel(['stimulus intensity (log cds/m²)'],'FontSize',8);ylabel('amplitude (V)','FontSize',8);

subplot(2,10,11:14); [AX,H1,H2] = plotyy(log10(Candela),((StimEndInd-StimStartInd)+uint32(IndexBFilt(:)))*1000/Fs,log10(Candela),1e6*DiffAmpBFilt,'plot');

title(sprintf('%s',['b-wave']),'FontSize',16);

xlabel(['stimulus intensity (log cds/m²)'],'FontSize',12);

set(get(AX(2),'Ylabel'),'String','amplitude (µV)'); set(H2,'LineStyle','-');

set(get(AX(1),'Ylabel'),'String','Latenz (msec)'); set(H1,'LineStyle',':'); set(AX(1),'YLim',[0 200]);

subplot(2,10,16:20); [AX,H1,H2] = plotyy(log10(Candela),((StimEndInd-StimStartInd)+uint32(IndexA(:)))*1000/Fs,log10(Candela),1e6*(DiffAmpAFirstMin*-1),'plot');

title(sprintf('%s',['a-wave']),'FontSize',16);

xlabel(['stimulus intensity (log cds/m²)'],'FontSize',12);

set(get(AX(2),'Ylabel'),'String','amplitude (µV)*-1'); set(H2,'LineStyle','-');

set(get(AX(1),'Ylabel'),'String','Latenz (msec)'); set(H1,'LineStyle',':'); set(AX(1),'YLim',[0 50]);

saveNJPG=fullfile(ispath,['sum\' name ' Sum.jpg']);

saveNFIG=fullfile(ispath,['sum\' name ' Sum.fig']);

print ('-djpeg','-r300',saveNJPG);

hgsave(h,saveNFIG);

end;

end;

if doAllPlot==1

if (StimulusDurScot>0)

h=figure;

subplot(2,2,1); plot(t(1:PlotTimePt),MaxMeanBSAllScop(:,(1:PlotTimePt)));

set(gca,'FontSize',12);title(sprintf('%s',[' ' isLastDir ' scot']),'FontSize',9);

xlabel(['time (msec) stimulus:' num2str(StimTime)],'FontSize',12);

ylabel('amplitude (V)','FontSize',12);

subplot(2,2,2); plot(log10(CandelaScot(:,:)),DiffAmpAmakrinScot(:,:),'-');

title(sprintf('%s',['oscillatory']),'FontSize',16);

xlabel(['stimulus intensity (log cds/m²)'],'FontSize',8);ylabel('amplitude (V)','FontSize',8);

subplot(2,2,3); plot(log10(CandelaScot(:,:)),DiffAmpBFiltScot(:,:),'-');

title(sprintf('%s',['b-wave']),'FontSize',16);

xlabel(['stimulus intensity (log cds/m²)'],'FontSize',12);

ylabel('amplitude (V)','FontSize',12);

subplot(2,2,4); plot(log10(CandelaScot(:,:)),DiffAmpAFirstMinScot(:,:),'-');

title(sprintf('%s',['a-wave']),'FontSize',16);

xlabel(['stimulus intensity (log cds/m²)'],'FontSize',12);

ylabel('amplitude (V)','FontSize',12);

set(gca,'YDir','reverse');

saveNJPG=fullfile(ispath,['sum\' isLastDir ' AllScot.jpg']);

print ('-djpeg','-r300',saveNJPG);

close(h);

end;

if (StimulusDurPhot>0)

h=figure;

subplot(2,2,1); plot(t(1:PlotTimePt),MaxMeanBSAllPhot(:,(1:PlotTimePt)));

set(gca,'FontSize',12);title(sprintf('%s',[' ' isLastDir ' phot']),'FontSize',9);

xlabel(['time (msec) stimulus:' num2str(StimTime)],'FontSize',12);

ylabel('amplitude (V)','FontSize',12);

subplot(2,2,2); plot(log10(CandelaPhot(:,:)),DiffAmpAmakrinPhot(:,:),'-');

title(sprintf('%s',['oscillatory']),'FontSize',16);

xlabel(['stimulus intensity (log cds/m²)'],'FontSize',8);ylabel('amplitude (V)','FontSize',8);

subplot(2,2,3); plot(log10(CandelaPhot(:,:)),DiffAmpBFiltPhot(:,:),'-');

title(sprintf('%s',['b-wave']),'FontSize',16);

xlabel(['stimulus intensity (log cds/m²)'],'FontSize',12);

ylabel('amplitude (V)','FontSize',12);

subplot(2,2,4); plot(log10(CandelaPhot(:,:)),DiffAmpAFirstMinPhot(:,:),'-');

title(sprintf('%s',['a-wave']),'FontSize',16);

xlabel(['stimulus intensity (log cds/m²)'],'FontSize',12);

ylabel('amplitude (V)','FontSize',12);

set(gca,'YDir','reverse');

saveNJPG=fullfile(ispath,['sum\' isLastDir ' AllPhot.jpg']);

print ('-djpeg','-r300',saveNJPG);

close(h);

end;

end;

end;

%end; %ispath

end;

end;

if(doXLSAccess==1) xlswrite('d:\resultAllERG',XLSArrayAccess,'Sheet1');end;
