# Supplementary figures and images for: Progressive axonopathy when oligodendrocytes lack the myelin protein CMTM5
Source: eLife. 2022 Mar 11;11:e75523. doi: 10.7554/eLife.75523 (PMC8916772; doi:10.7554/eLife.75523)

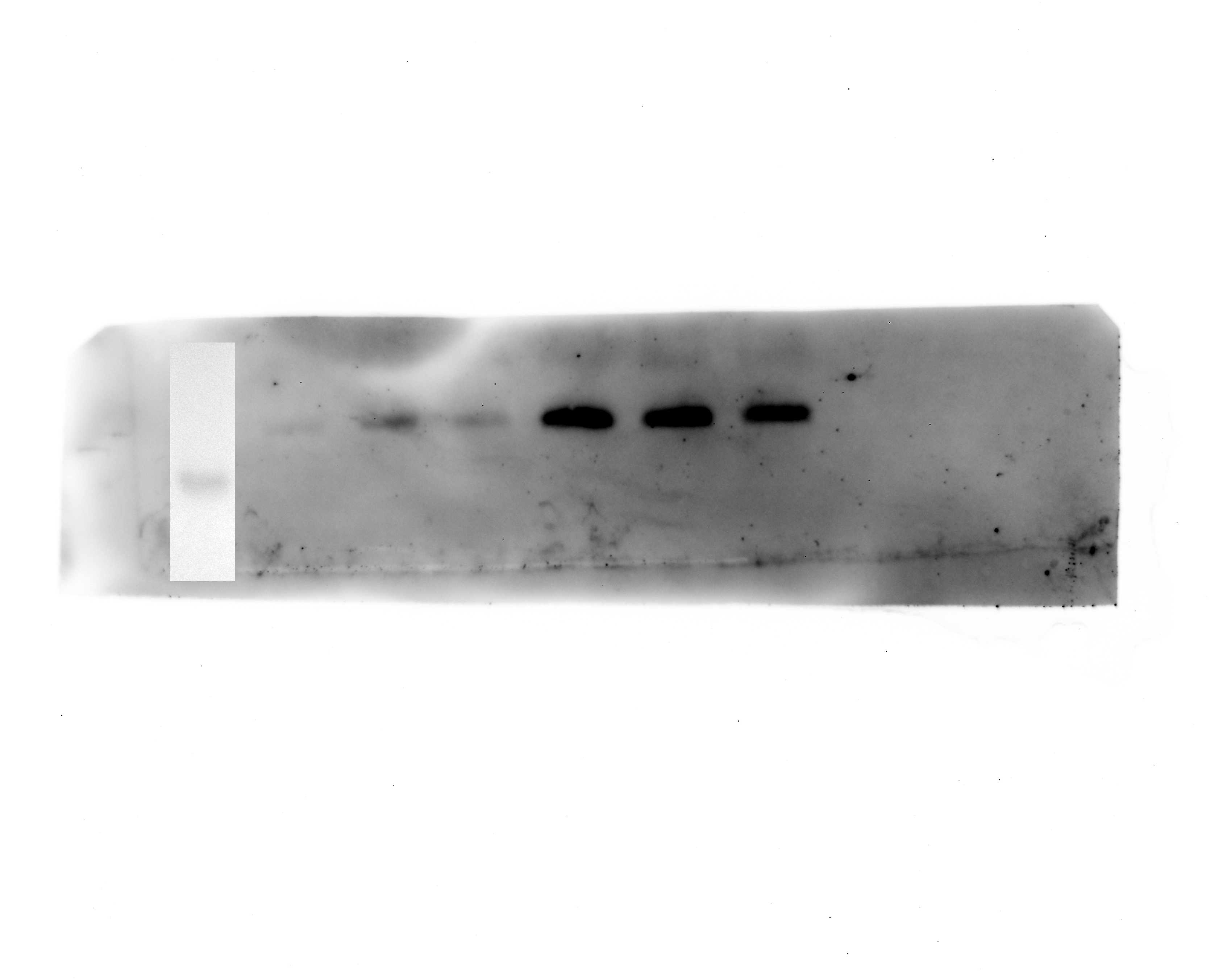

Supplement: Source data 1. [file elife-75523-data1.zip › Buscham Source Data Blots/Figure 1A Blot source data/Figure 1A CMTM5.tif]

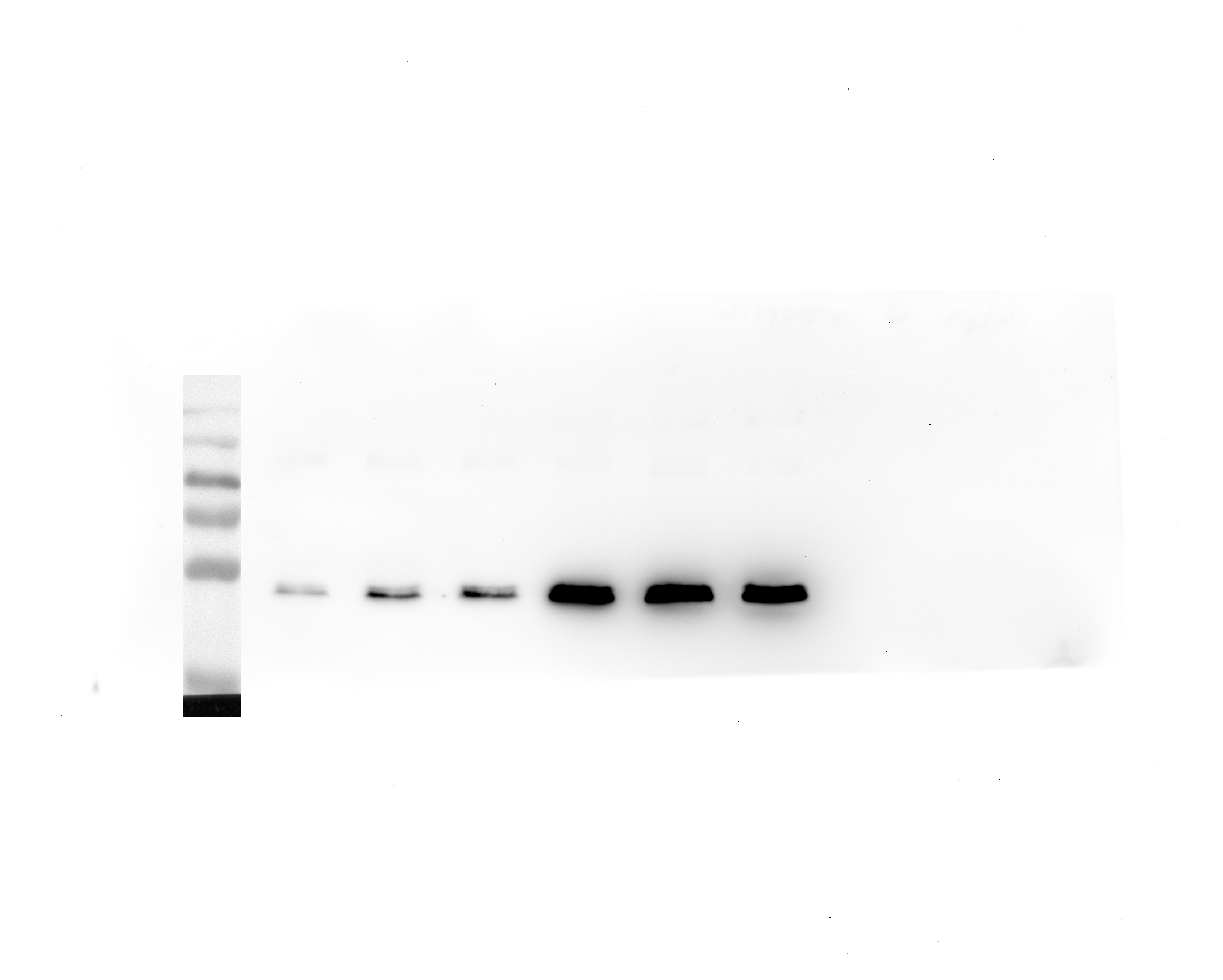

Supplement: Source data 1. [file elife-75523-data1.zip › Buscham Source Data Blots/Figure 1A Blot source data/Figure 1A CNP.tif]

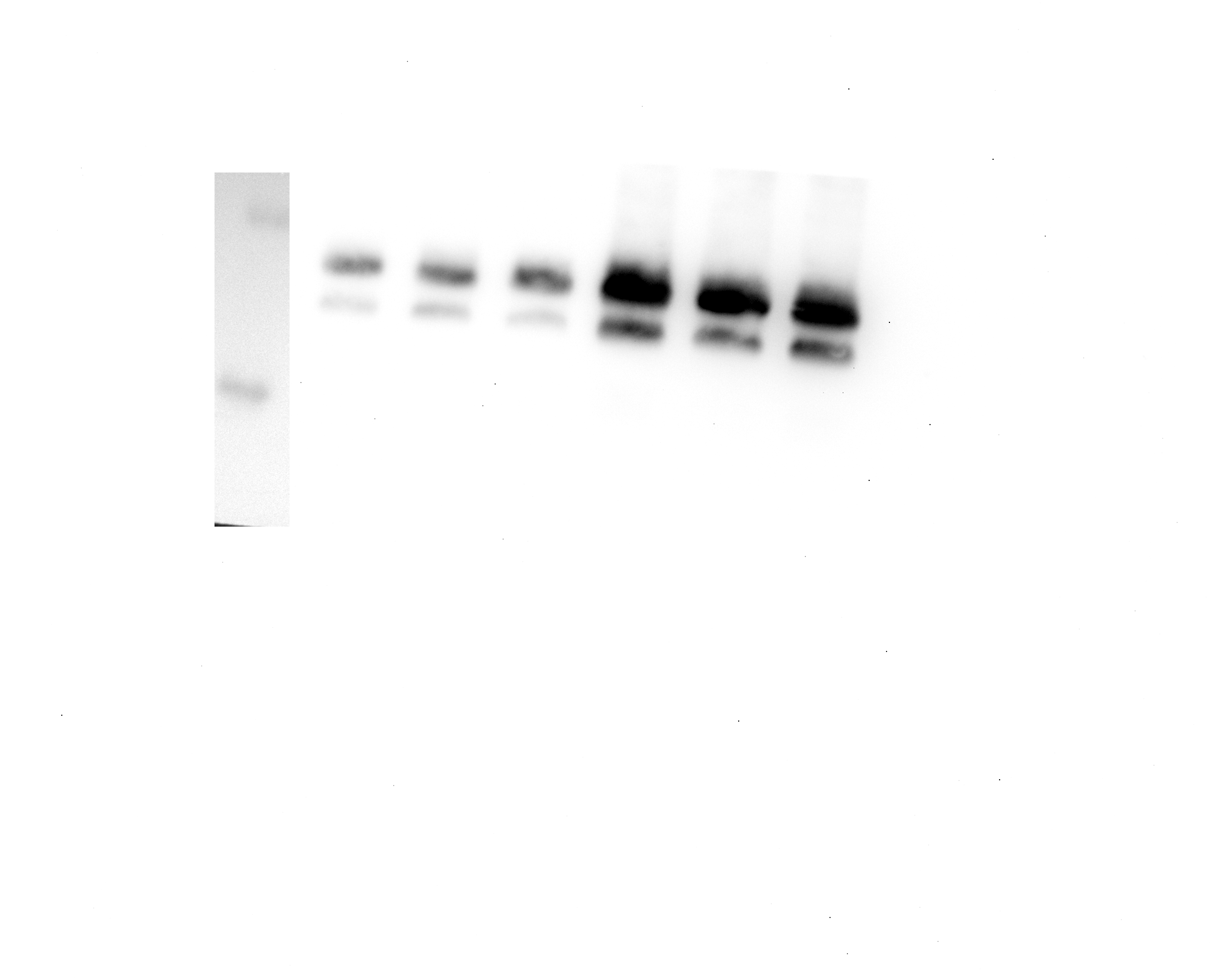

Supplement: Source data 1. [file elife-75523-data1.zip › Buscham Source Data Blots/Figure 1A Blot source data/Figure 1A PLP1.tif]

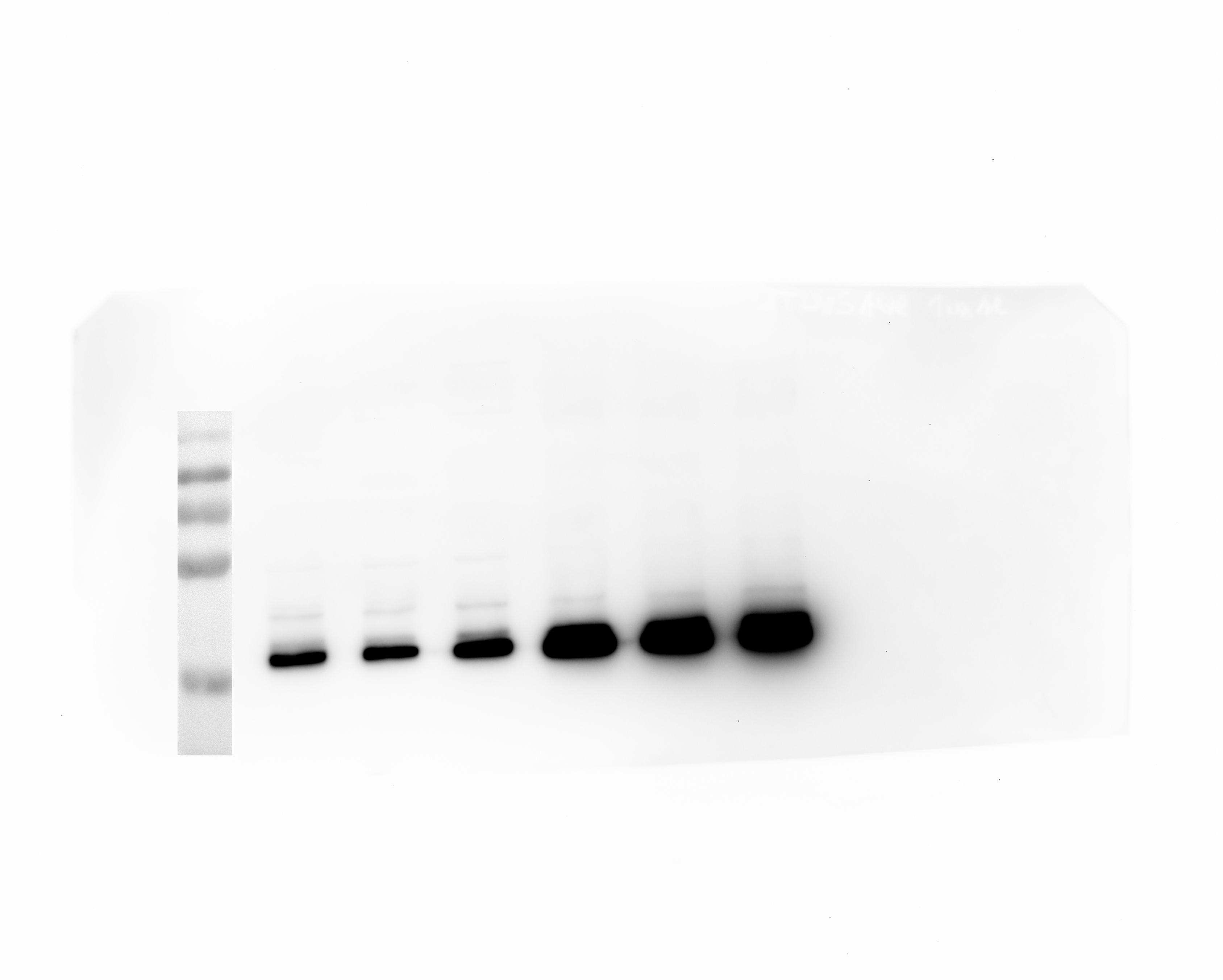

Supplement: Source data 1. [file elife-75523-data1.zip › Buscham Source Data Blots/Figure 1A Blot source data/Figure 1A SIRT2.tif]

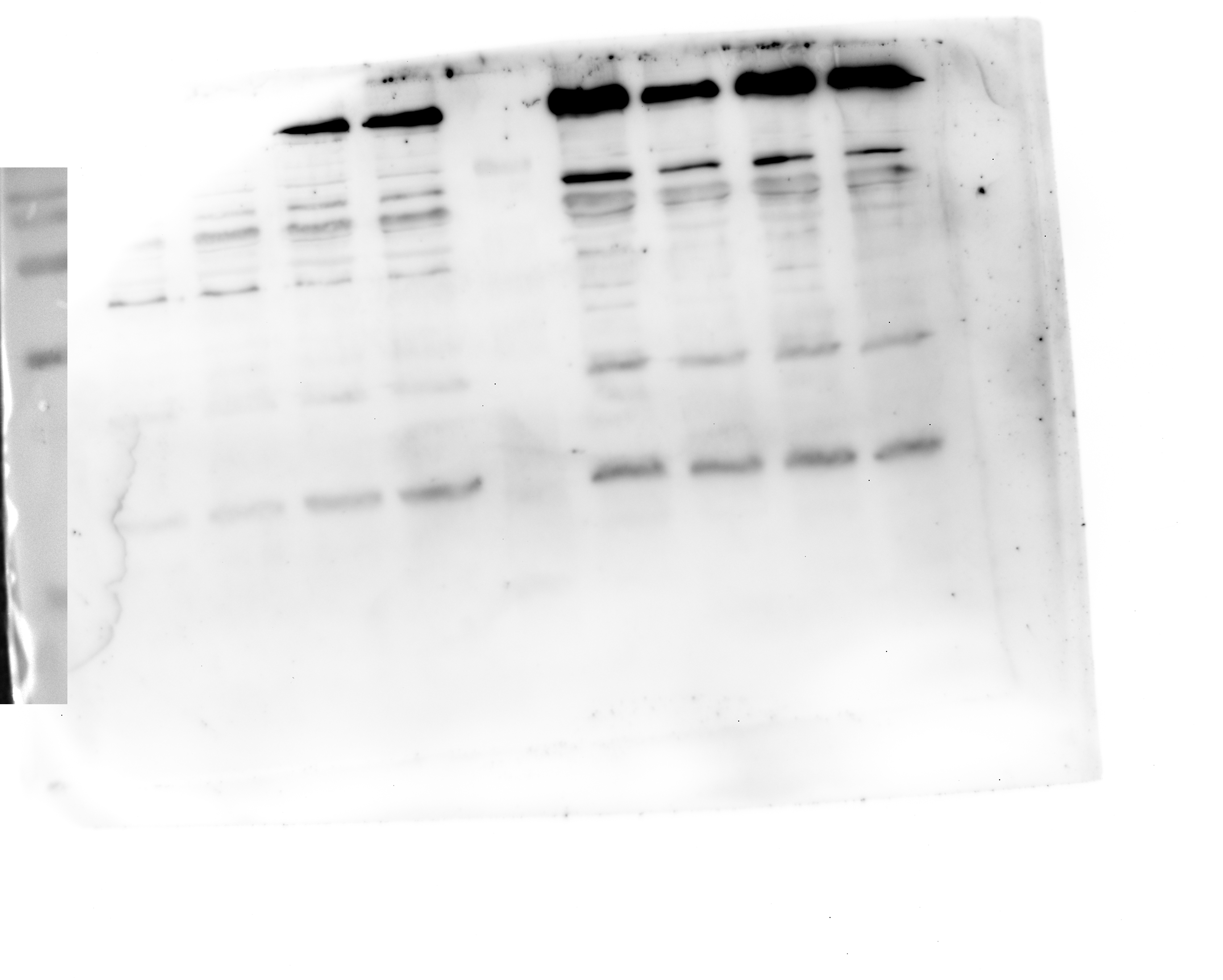

Supplement: Source data 1. [file elife-75523-data1.zip › Buscham Source Data Blots/Figure 1C Blot source data/Figure 1C CMTM5 P15-P24 and 6m-24m.tif]

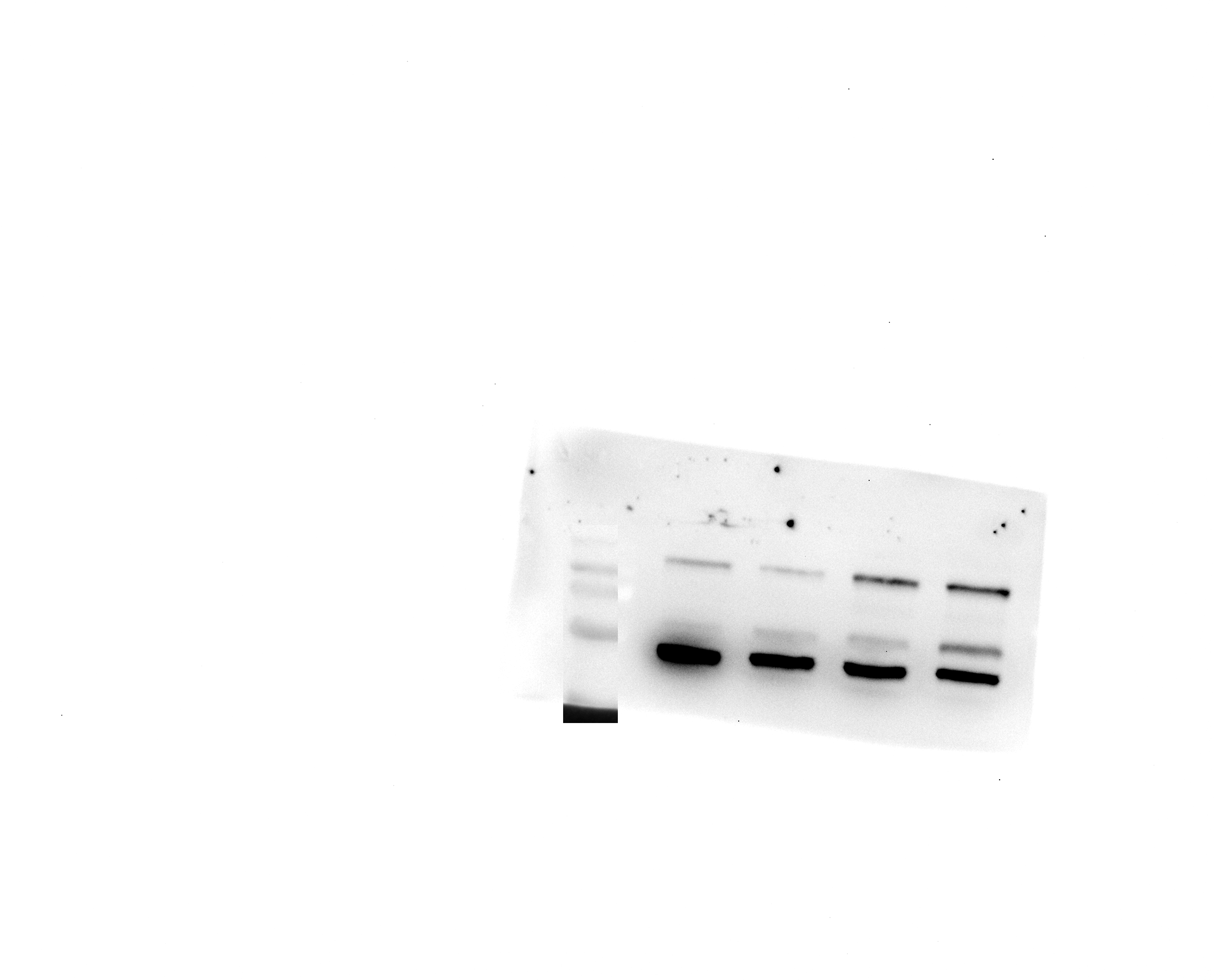

Supplement: Source data 1. [file elife-75523-data1.zip › Buscham Source Data Blots/Figure 1C Blot source data/Figure 1C CNP 6m-24m.tif]

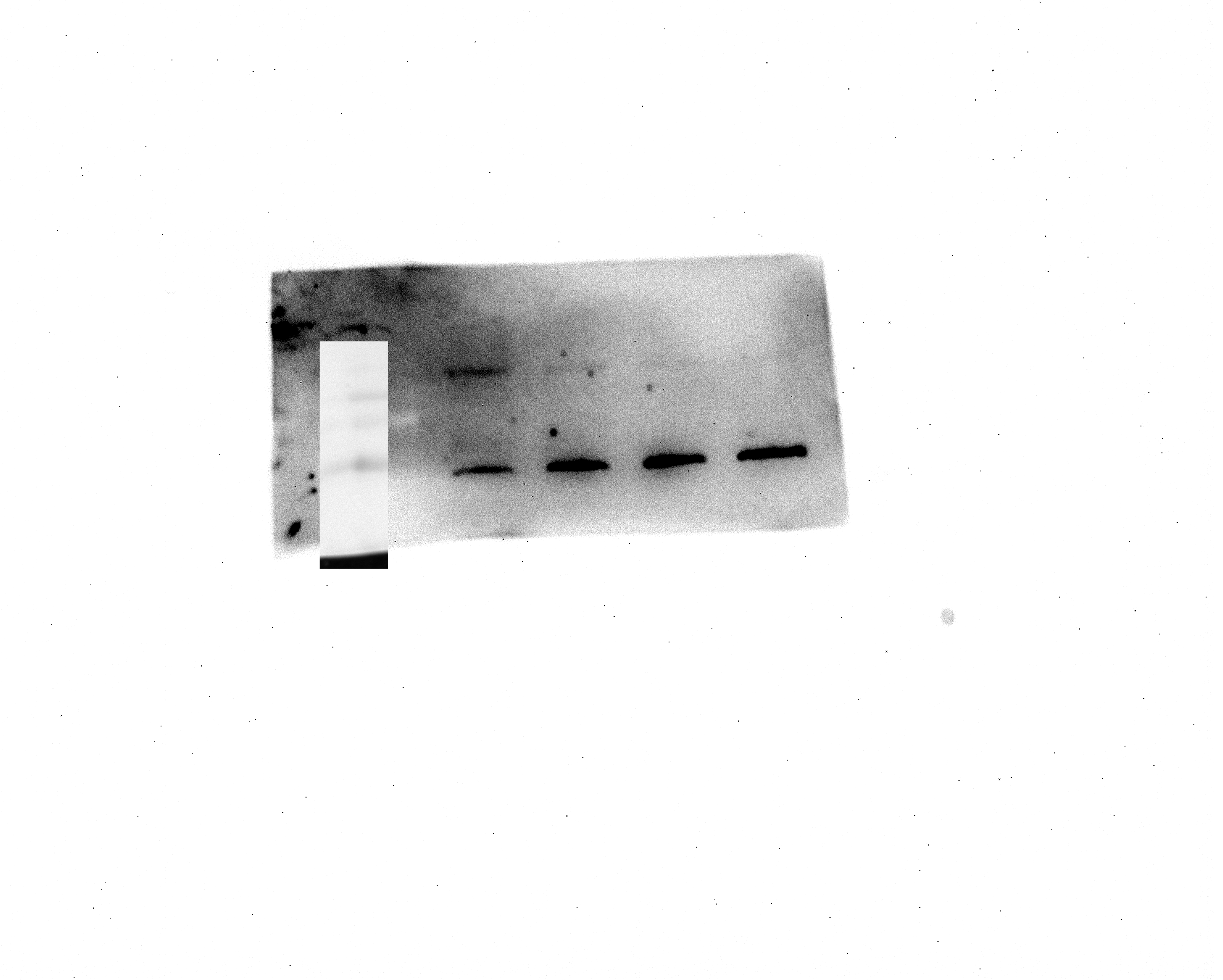

Supplement: Source data 1. [file elife-75523-data1.zip › Buscham Source Data Blots/Figure 1C Blot source data/Figure 1C CNP P15-P24.tif]

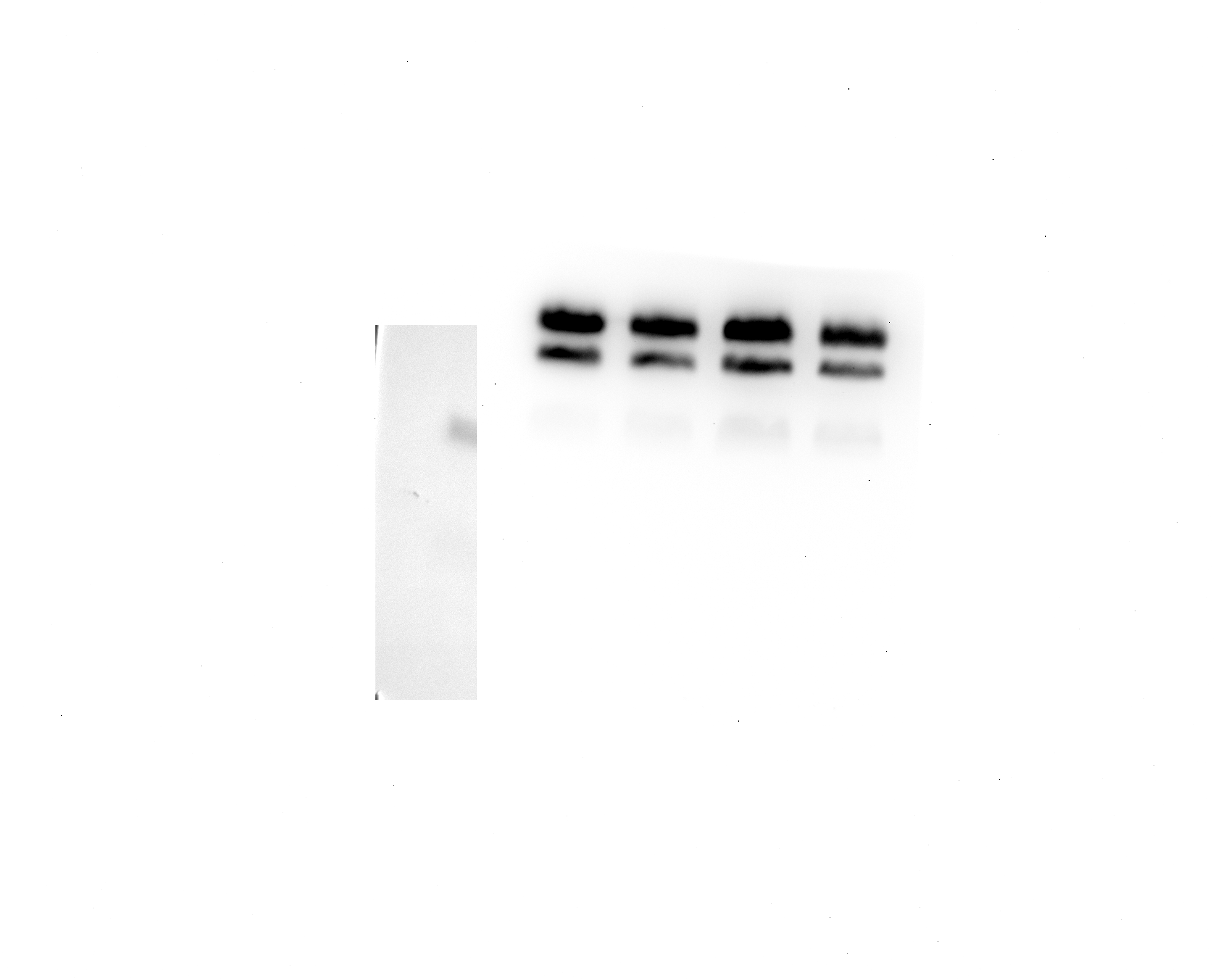

Supplement: Source data 1. [file elife-75523-data1.zip › Buscham Source Data Blots/Figure 1C Blot source data/Figure 1C PLP 6m-24m.tif]

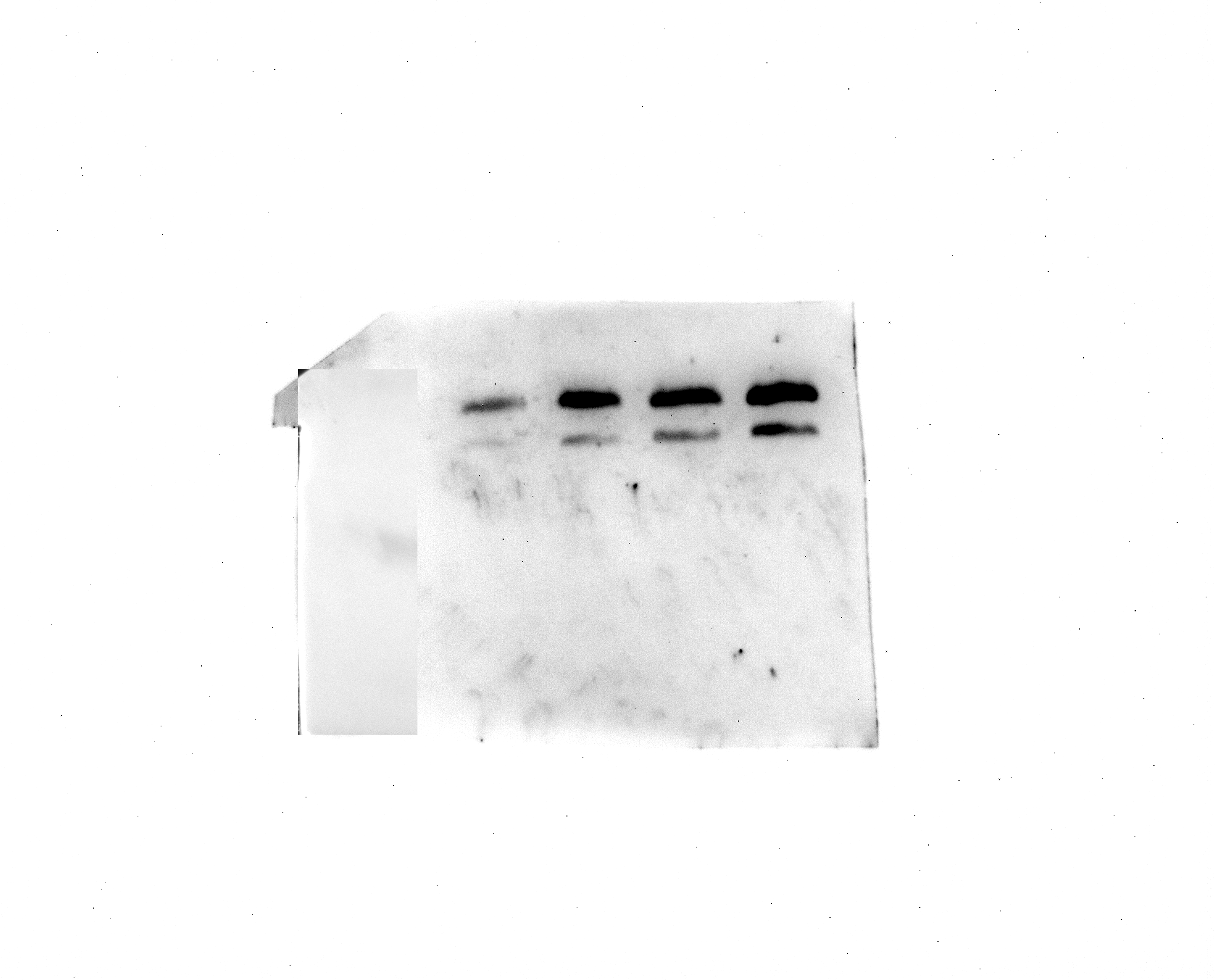

Supplement: Source data 1. [file elife-75523-data1.zip › Buscham Source Data Blots/Figure 1C Blot source data/Figure 1C PLP P15-P24.tif]

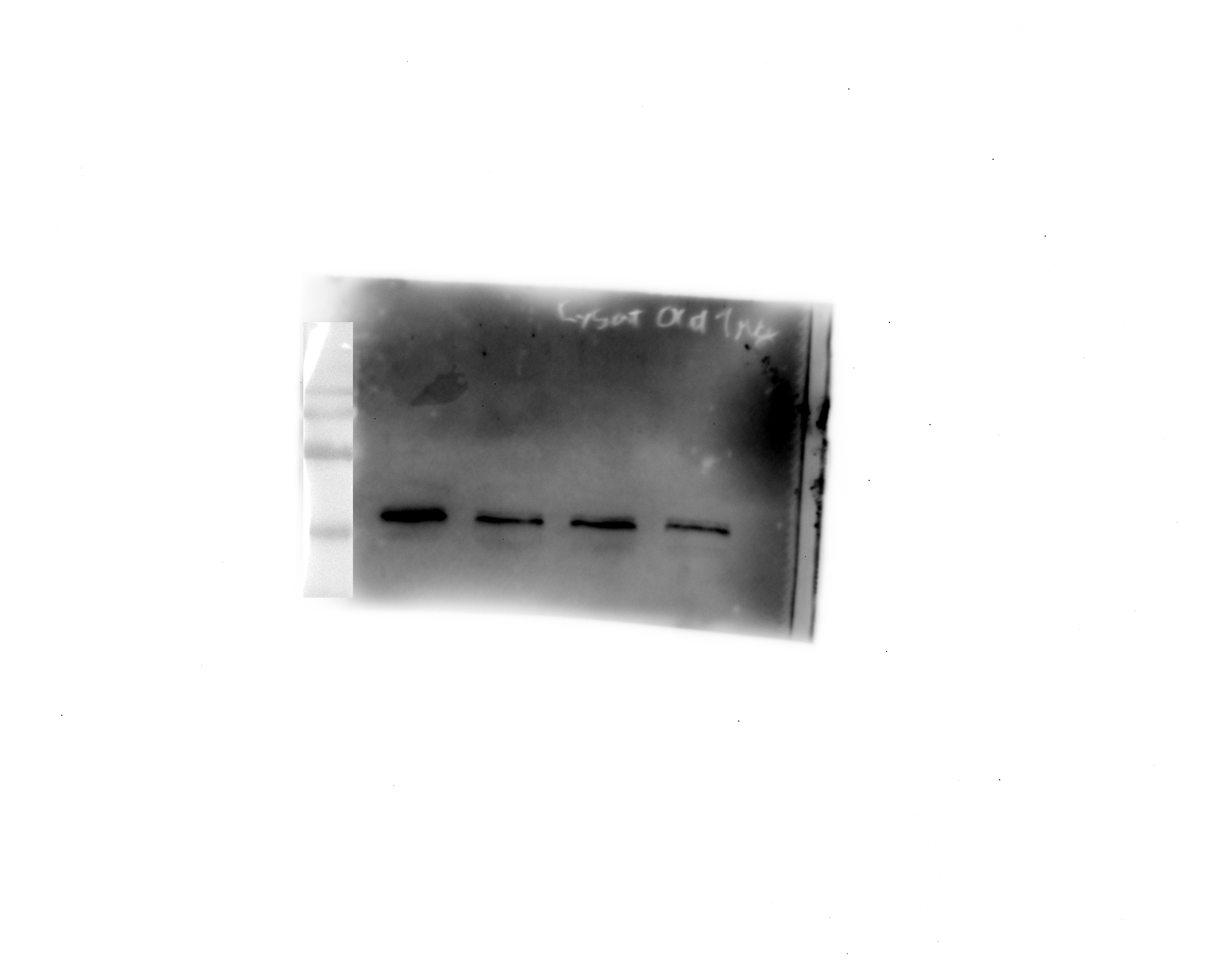

Supplement: Source data 1. [file elife-75523-data1.zip › Buscham Source Data Blots/Figure 1C Blot source data/Figure 1C SIRT2 6m-24m.tif]

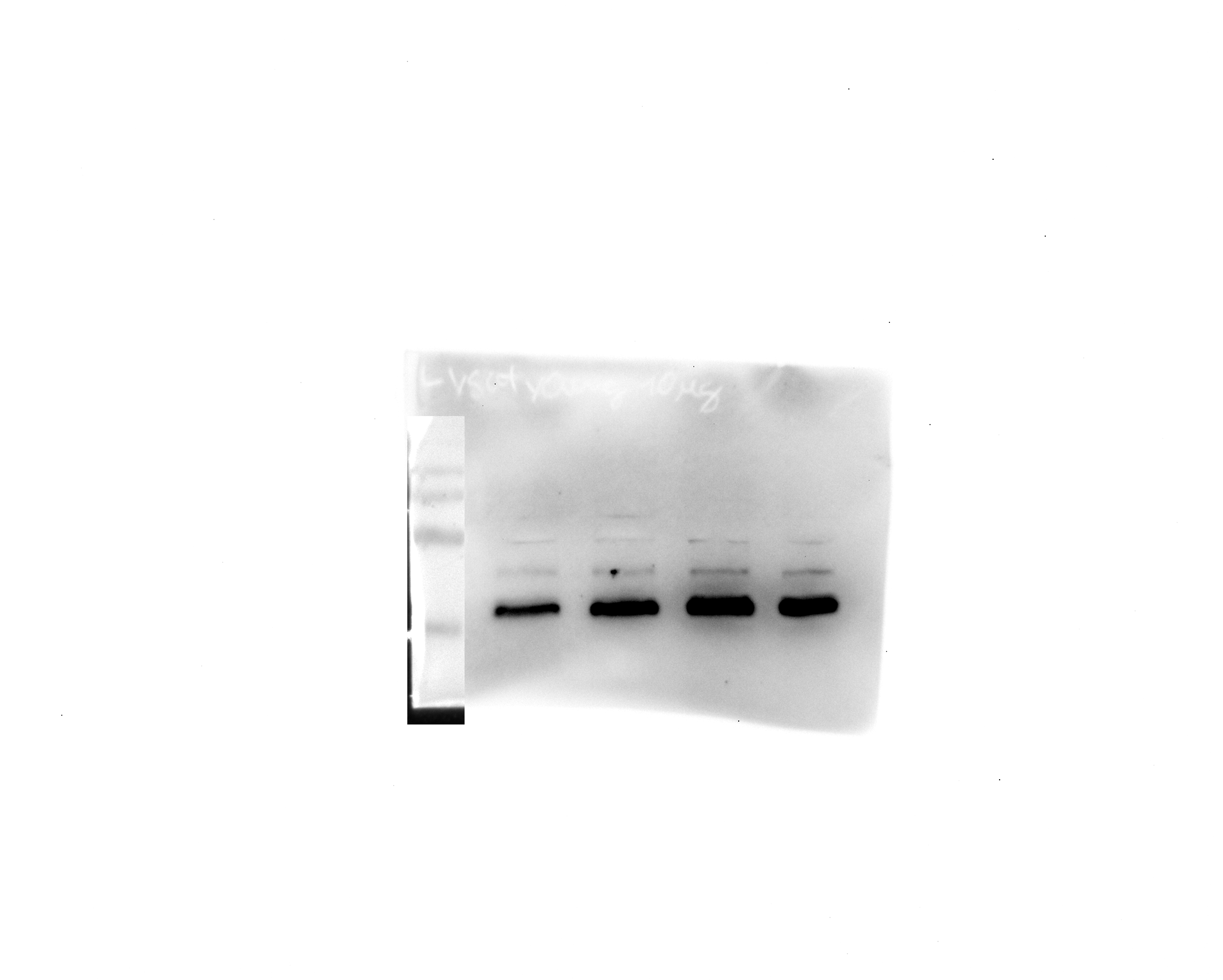

Supplement: Source data 1. [file elife-75523-data1.zip › Buscham Source Data Blots/Figure 1C Blot source data/Figure 1C SIRT2 P15-P24.tif]

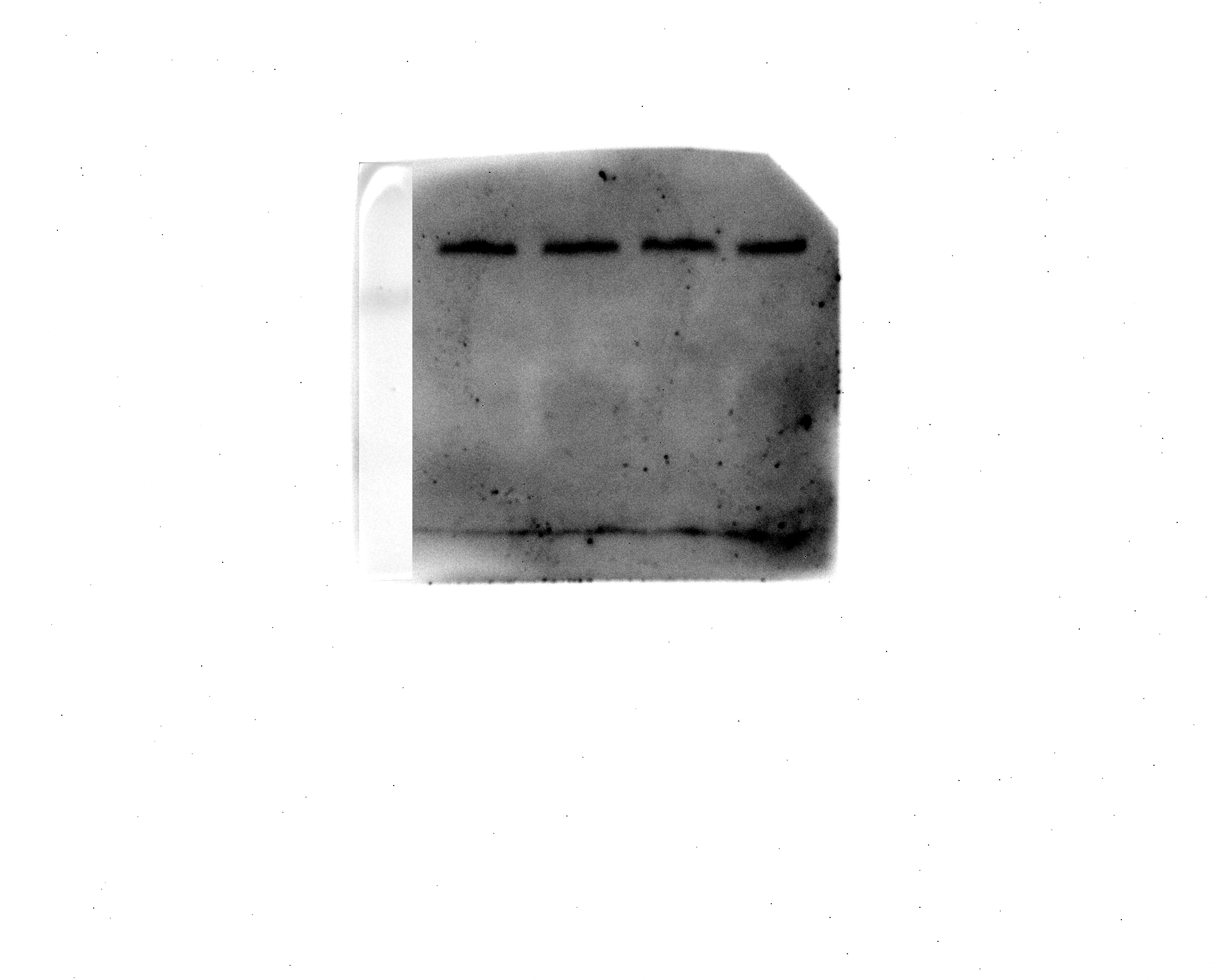

Supplement: Source data 1. [file elife-75523-data1.zip › Buscham Source Data Blots/Figure 1D Blot source data/Figure 1D CMTM5 6m-24m.tif]

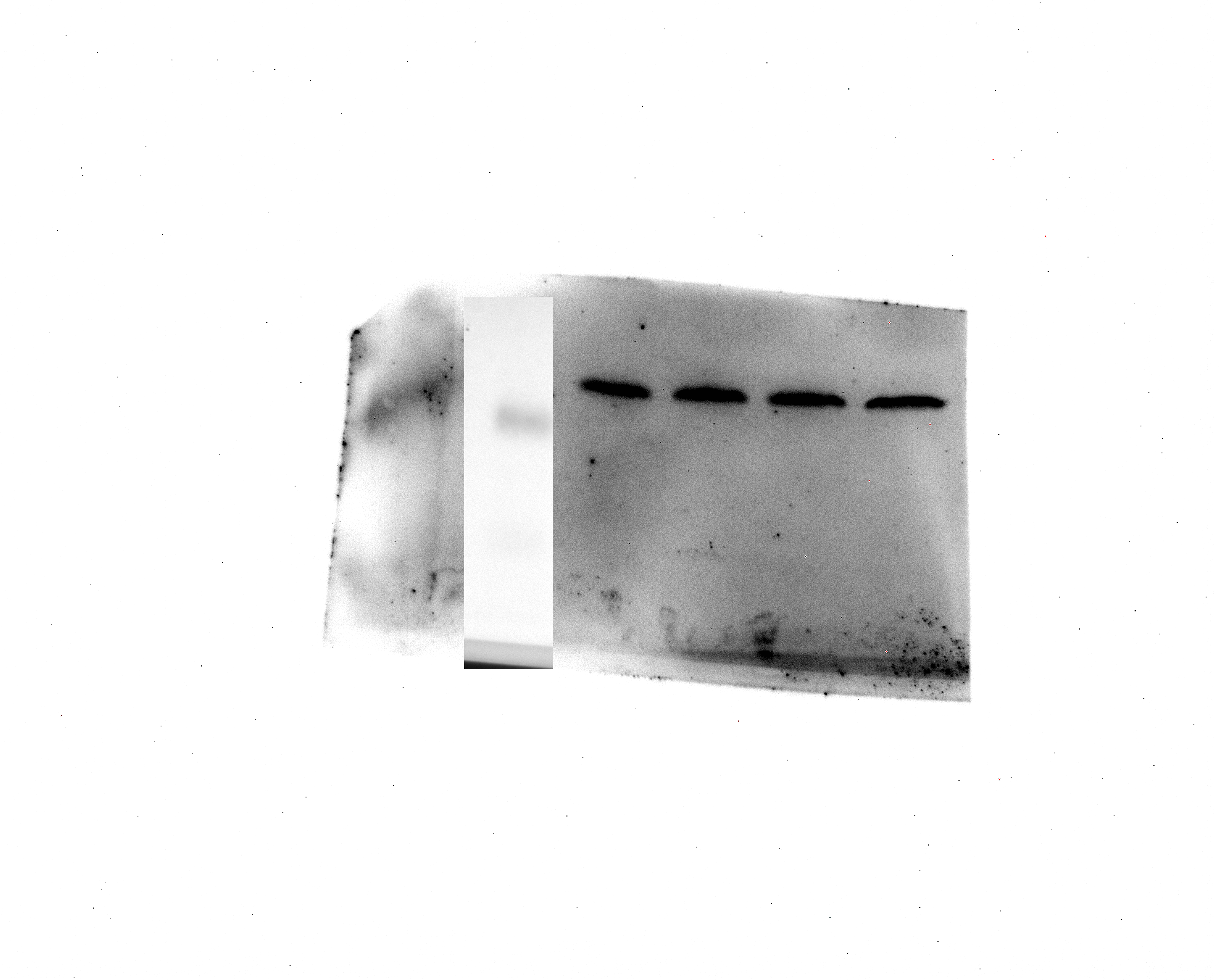

Supplement: Source data 1. [file elife-75523-data1.zip › Buscham Source Data Blots/Figure 1D Blot source data/Figure 1D CMTM5 P15-P24.tif]

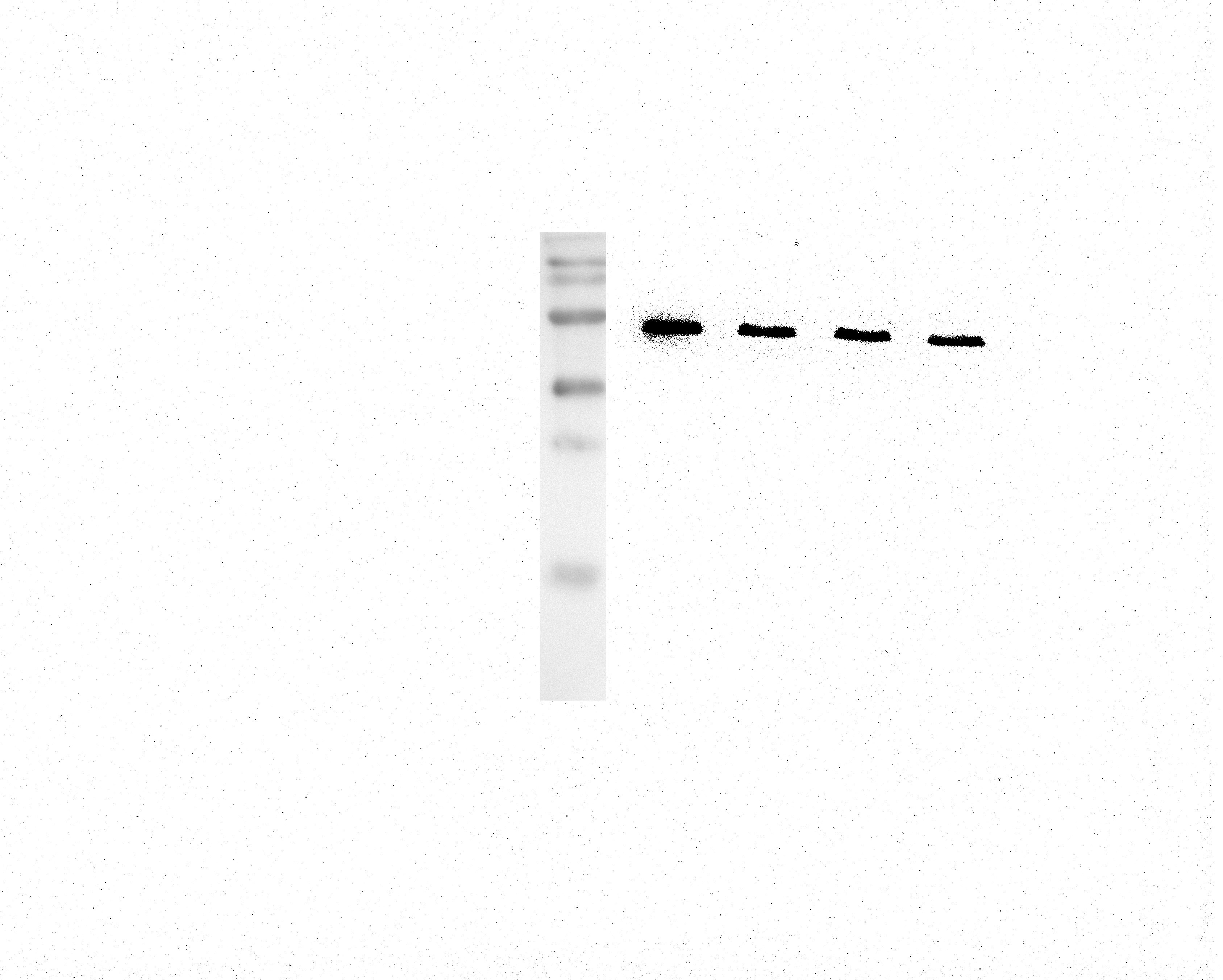

Supplement: Source data 1. [file elife-75523-data1.zip › Buscham Source Data Blots/Figure 1D Blot source data/Figure 1D CNP 6m-24m.tif]

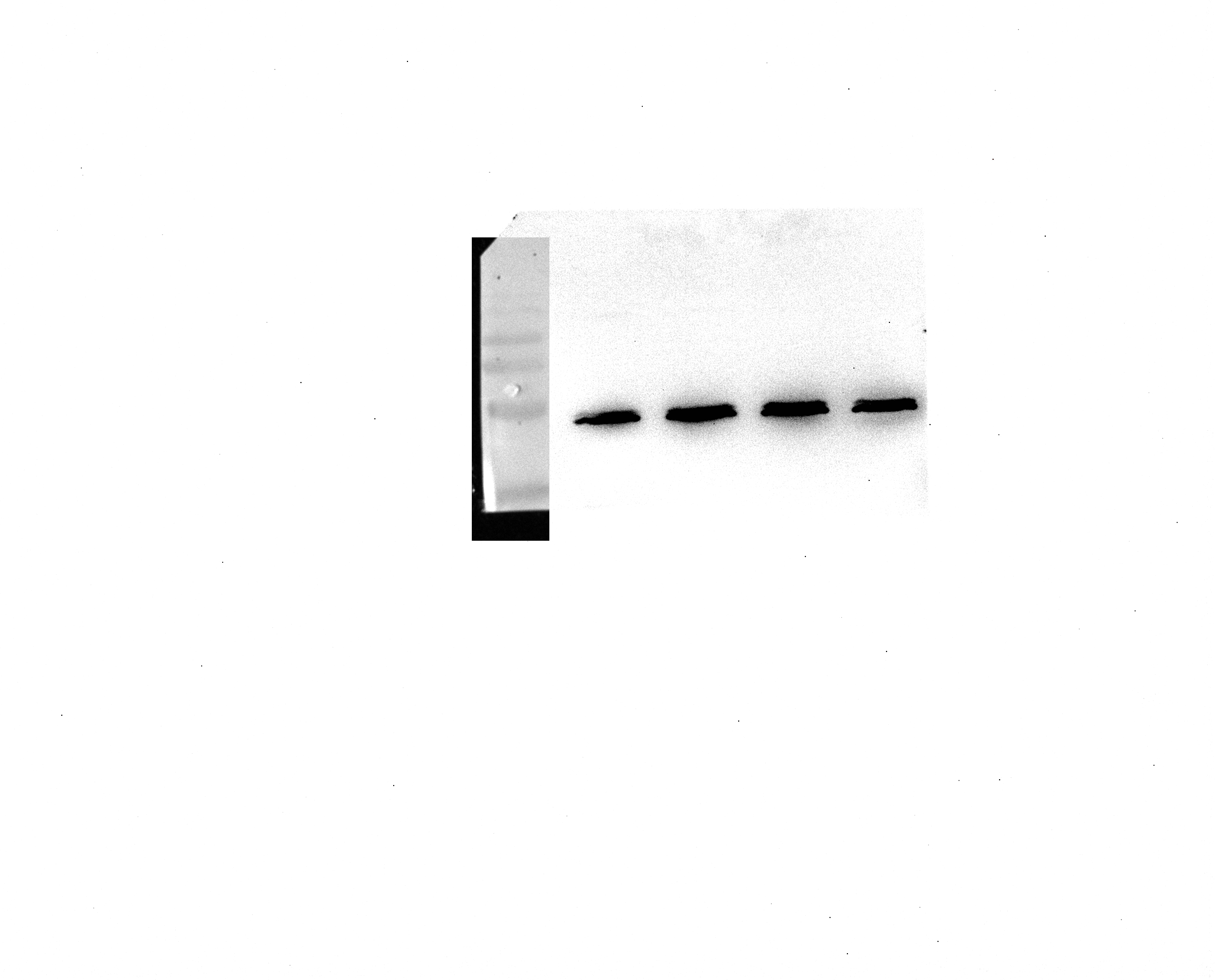

Supplement: Source data 1. [file elife-75523-data1.zip › Buscham Source Data Blots/Figure 1D Blot source data/Figure 1D CNP P15-P24.tif]

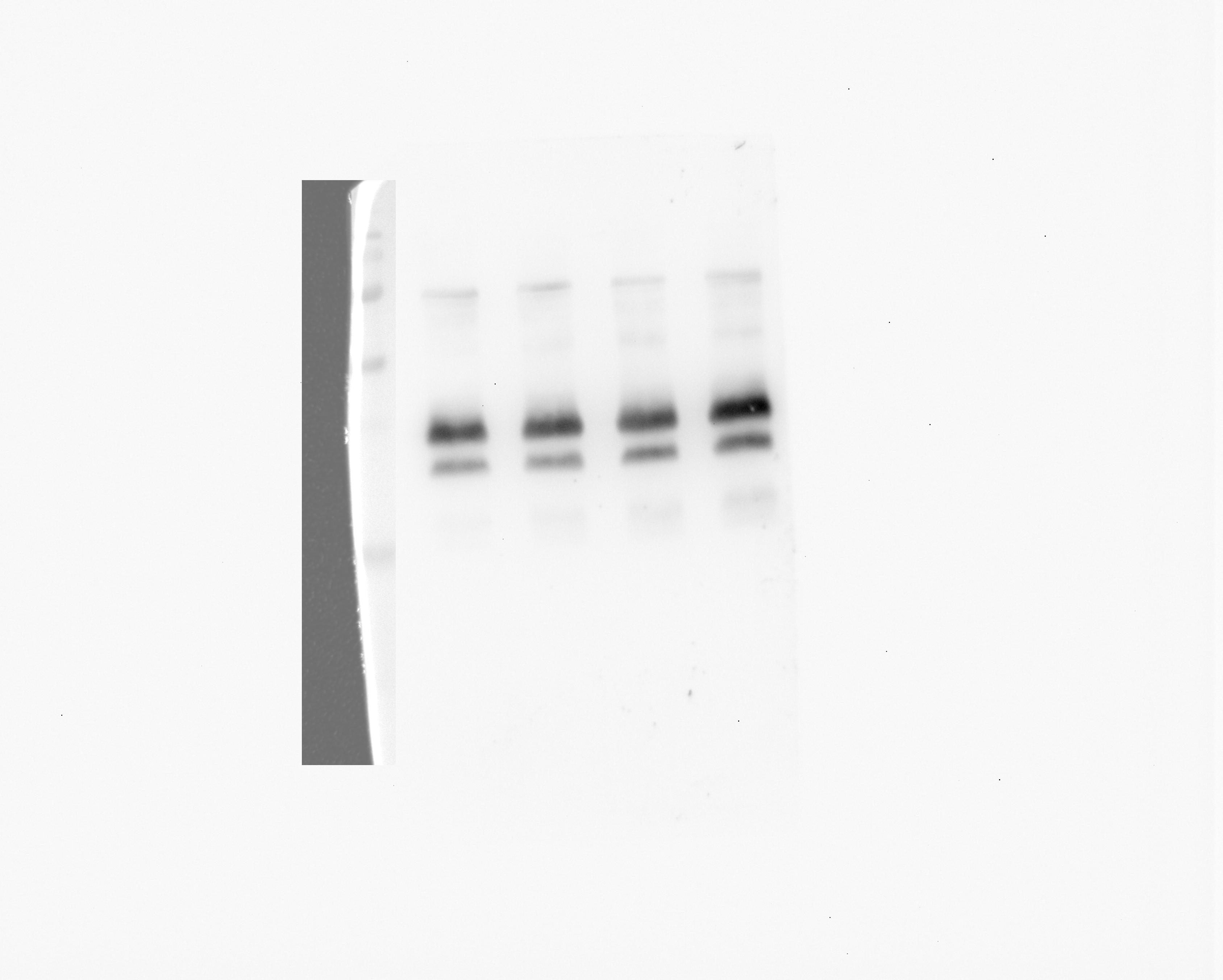

Supplement: Source data 1. [file elife-75523-data1.zip › Buscham Source Data Blots/Figure 1D Blot source data/Figure 1D PLP 6m-24m.tif]

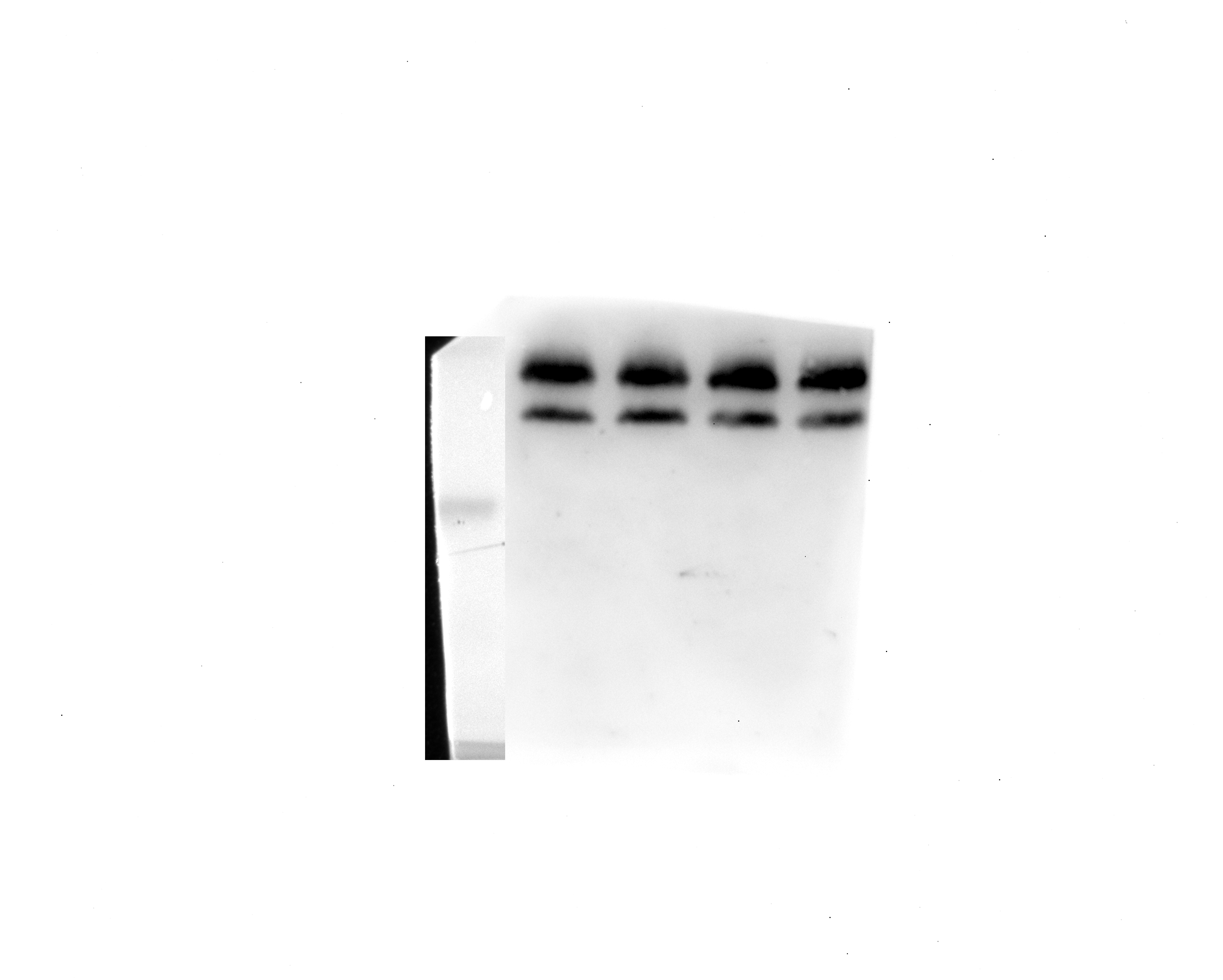

Supplement: Source data 1. [file elife-75523-data1.zip › Buscham Source Data Blots/Figure 1D Blot source data/Figure 1D PLP P15-P24.tif]

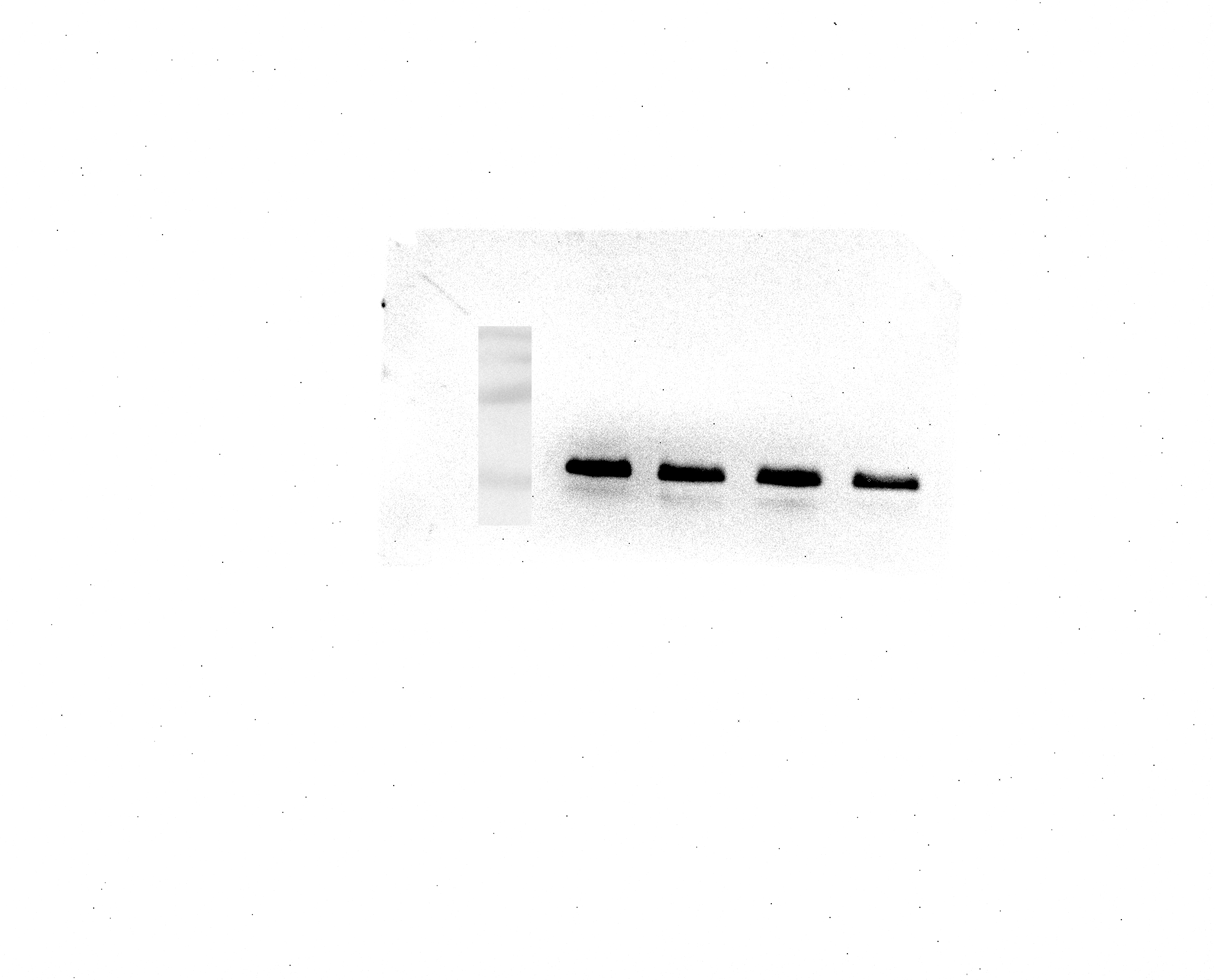

Supplement: Source data 1. [file elife-75523-data1.zip › Buscham Source Data Blots/Figure 1D Blot source data/Figure 1D SIRT2 6m-24m.tif]

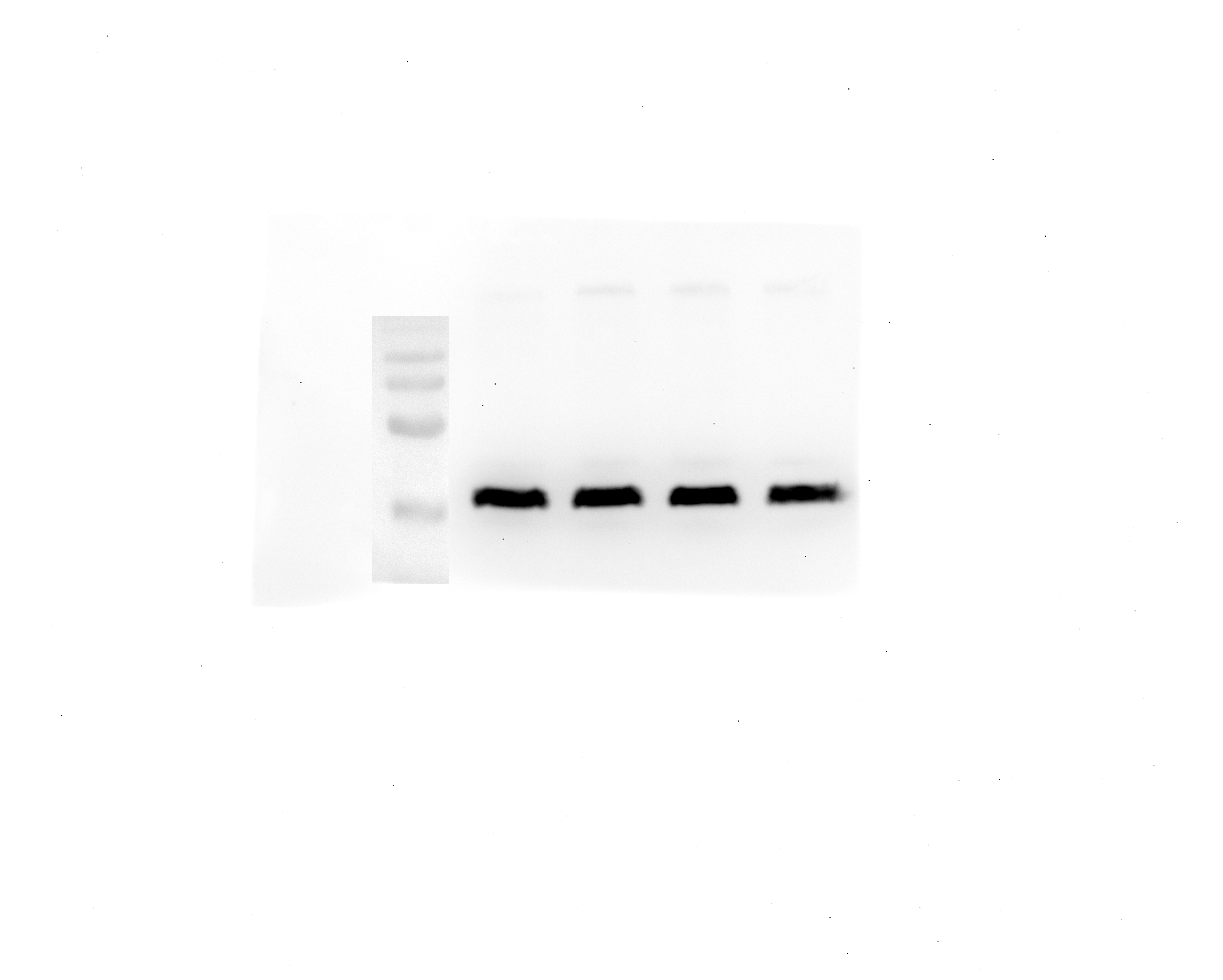

Supplement: Source data 1. [file elife-75523-data1.zip › Buscham Source Data Blots/Figure 1D Blot source data/Figure 1D SIRT2 P15-P24.tif]

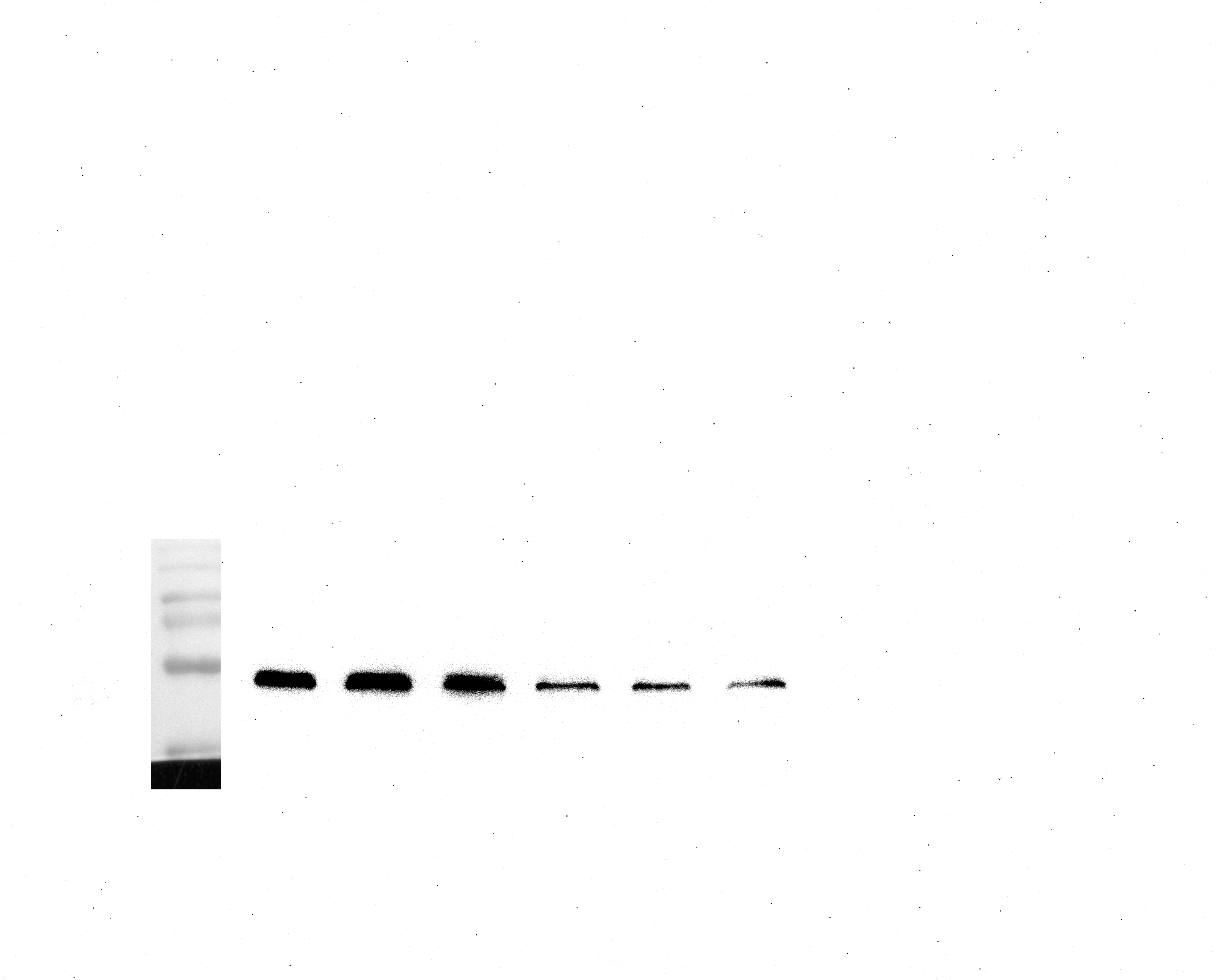

Supplement: Source data 1. [file elife-75523-data1.zip › Buscham Source Data Blots/Figure 2A Blot source data/Figure 2A CNP.tif]

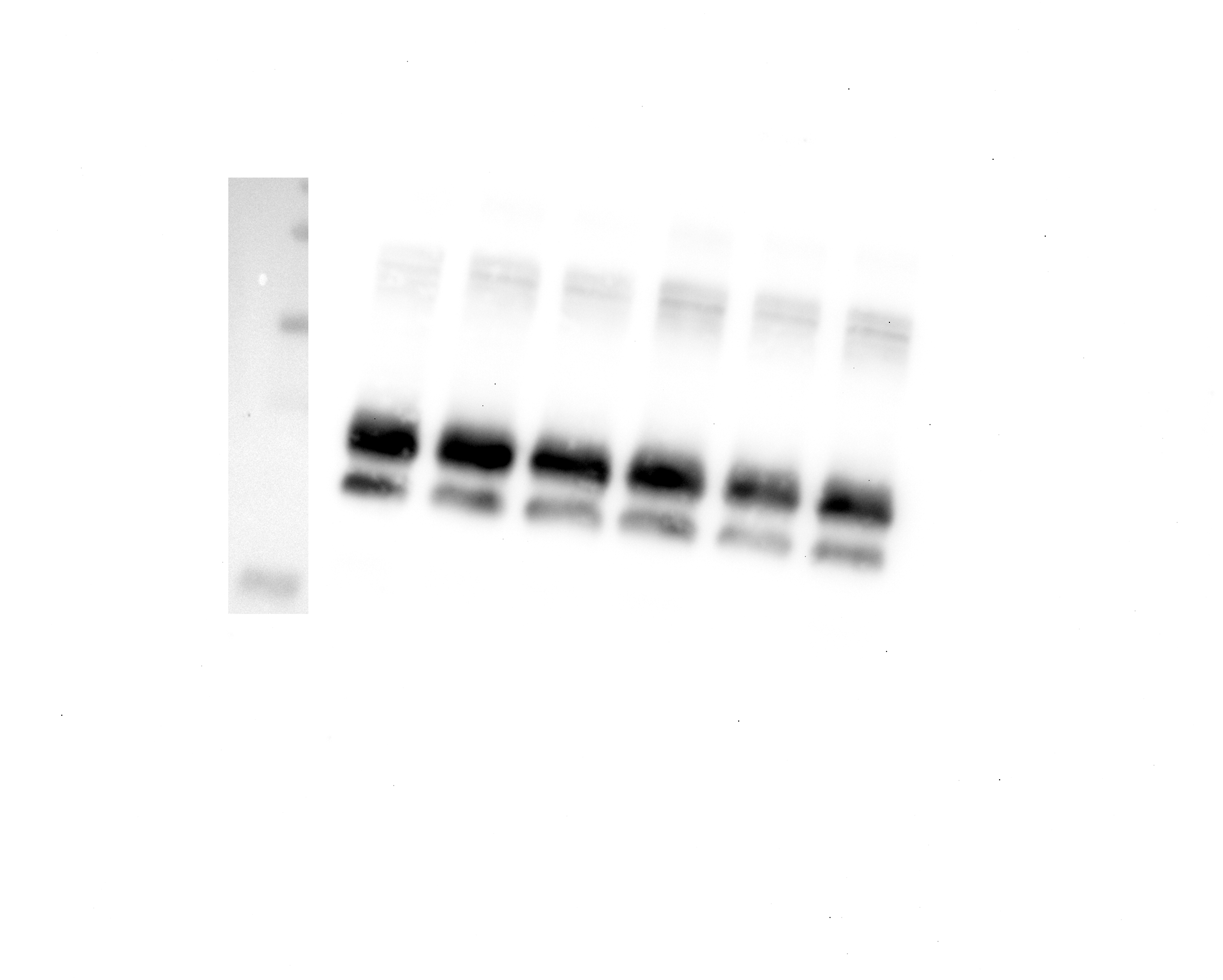

Supplement: Source data 1. [file elife-75523-data1.zip › Buscham Source Data Blots/Figure 2A Blot source data/Figure 2A PLP.tif]

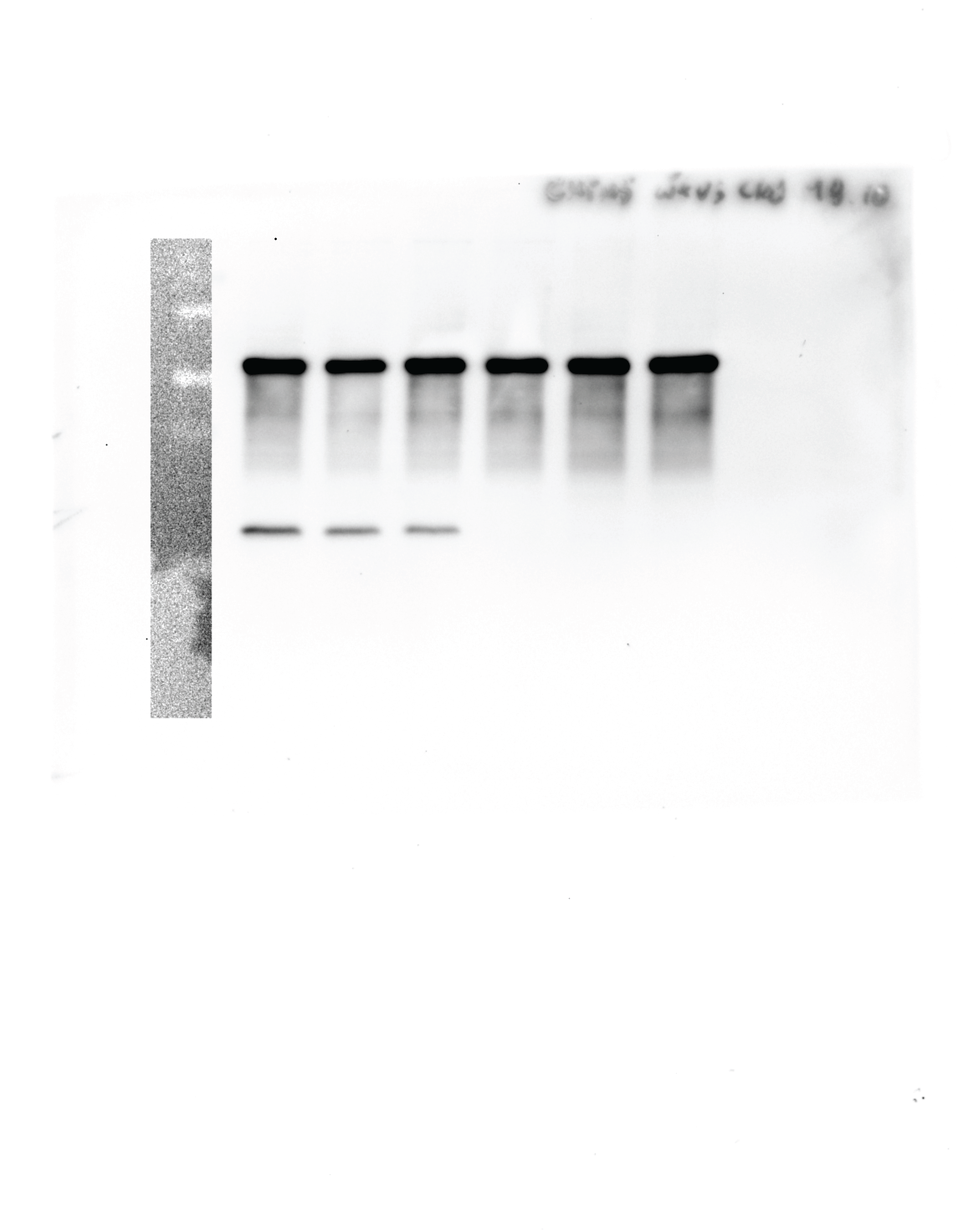

Supplement: Source data 1. [file elife-75523-data1.zip › Buscham Source Data Blots/Figure 2A Blot source data/Figure 2A SIRT2 and CMTM5.tif]

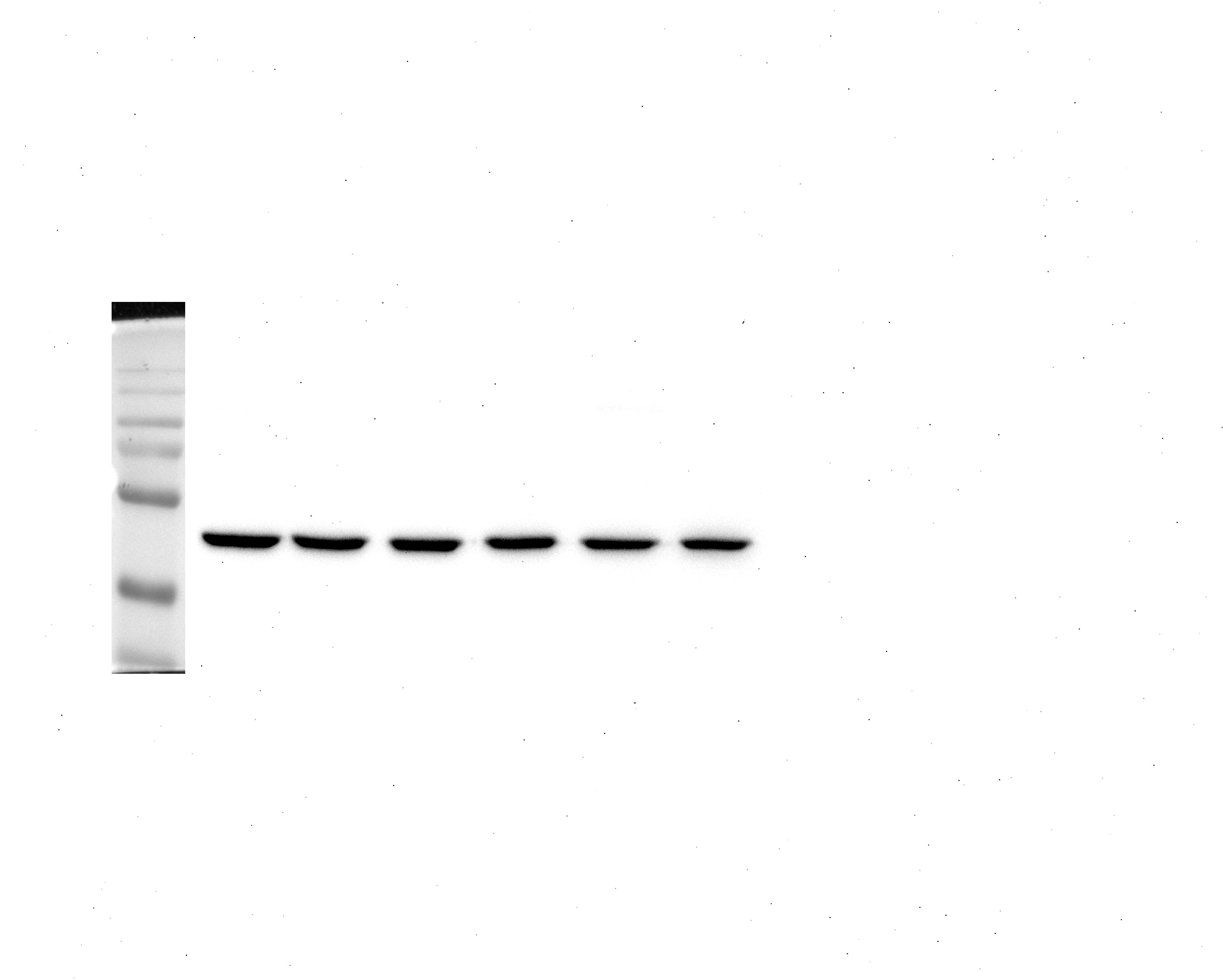

Supplement: Source data 1. [file elife-75523-data1.zip › Buscham Source Data Blots/Figure 2O Blot source data/Figure 2O actin.tif]

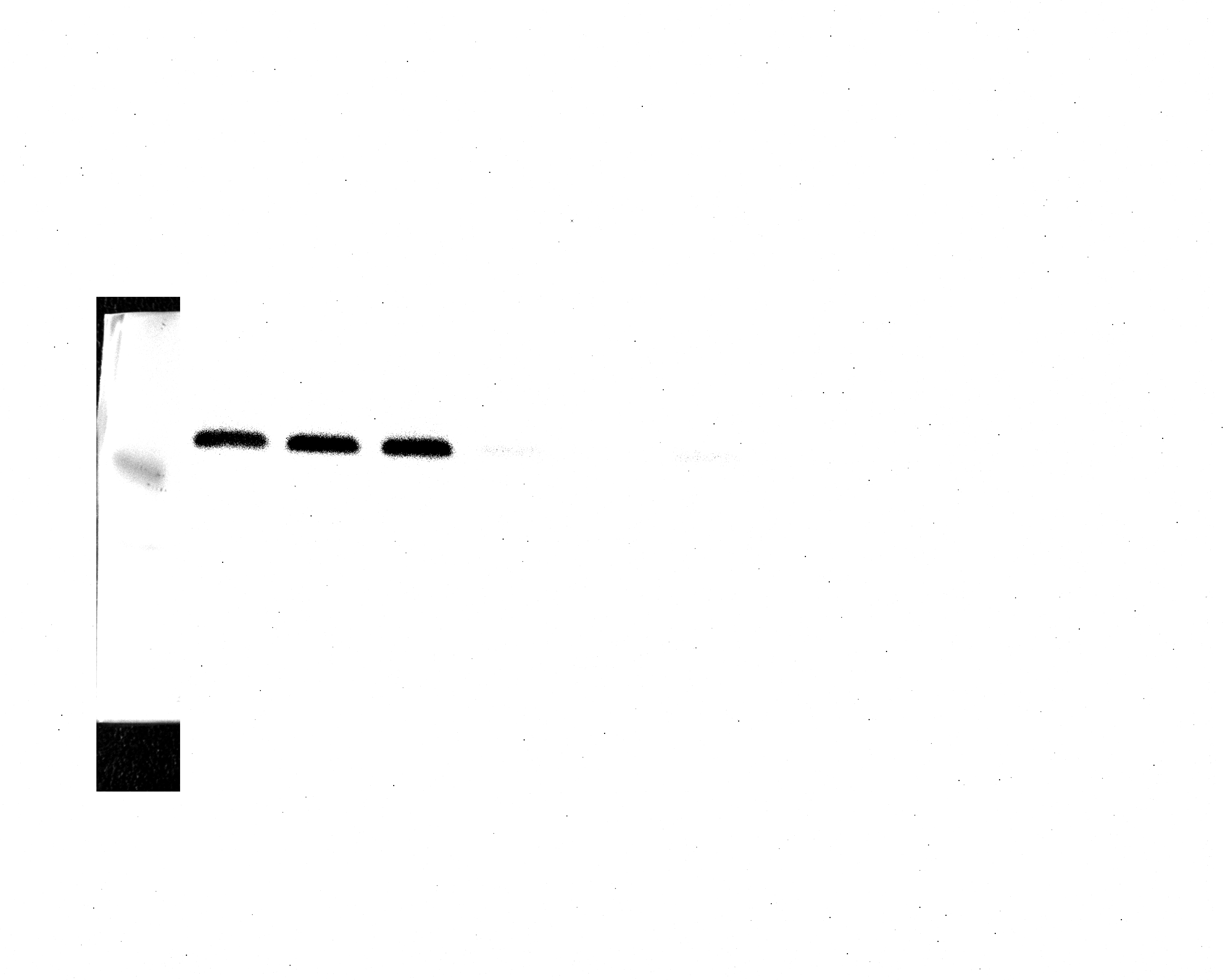

Supplement: Source data 1. [file elife-75523-data1.zip › Buscham Source Data Blots/Figure 2O Blot source data/Figure 2O CMTM5.tif]

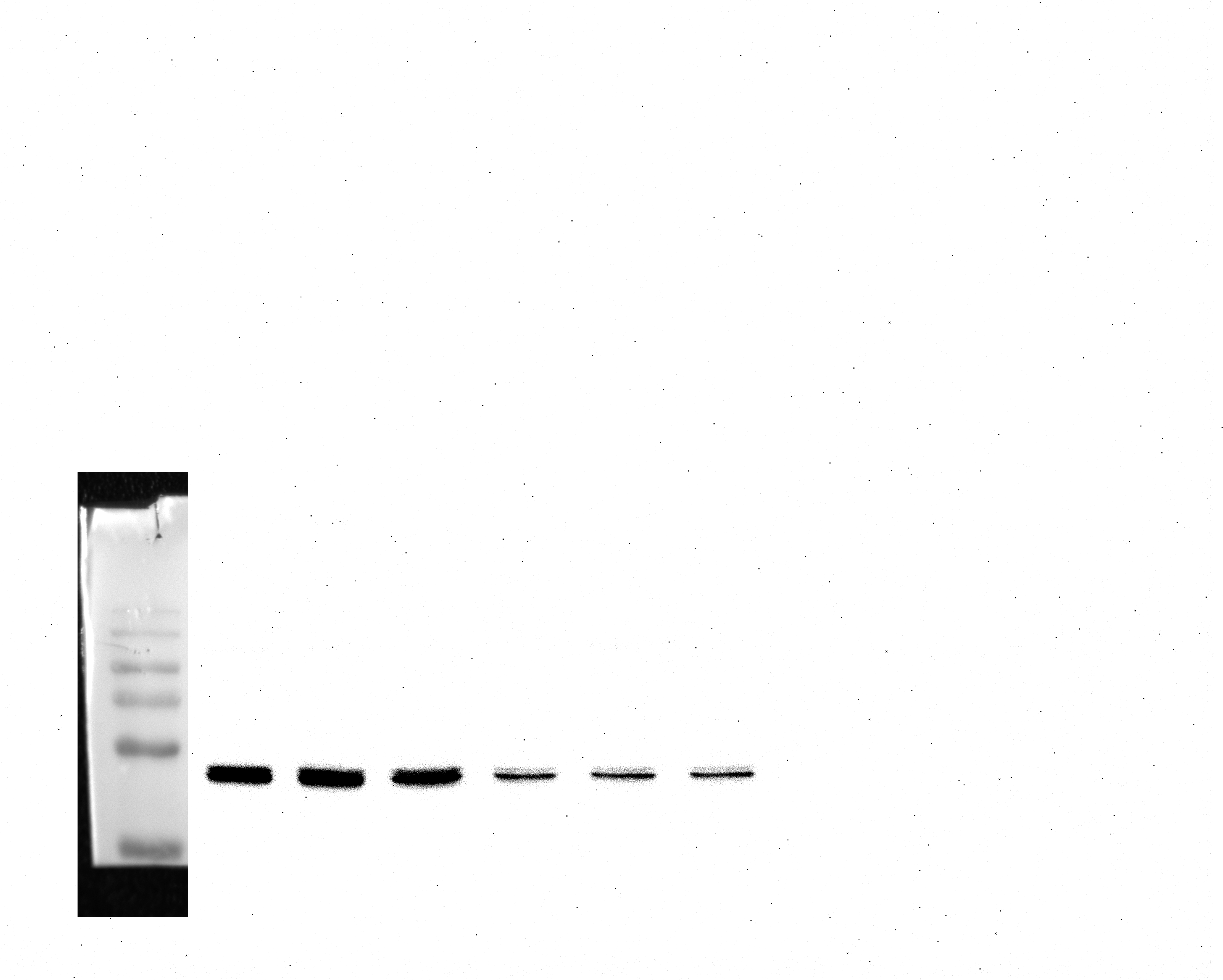

Supplement: Source data 1. [file elife-75523-data1.zip › Buscham Source Data Blots/Figure 2O Blot source data/Figure 2O CNP.tif]

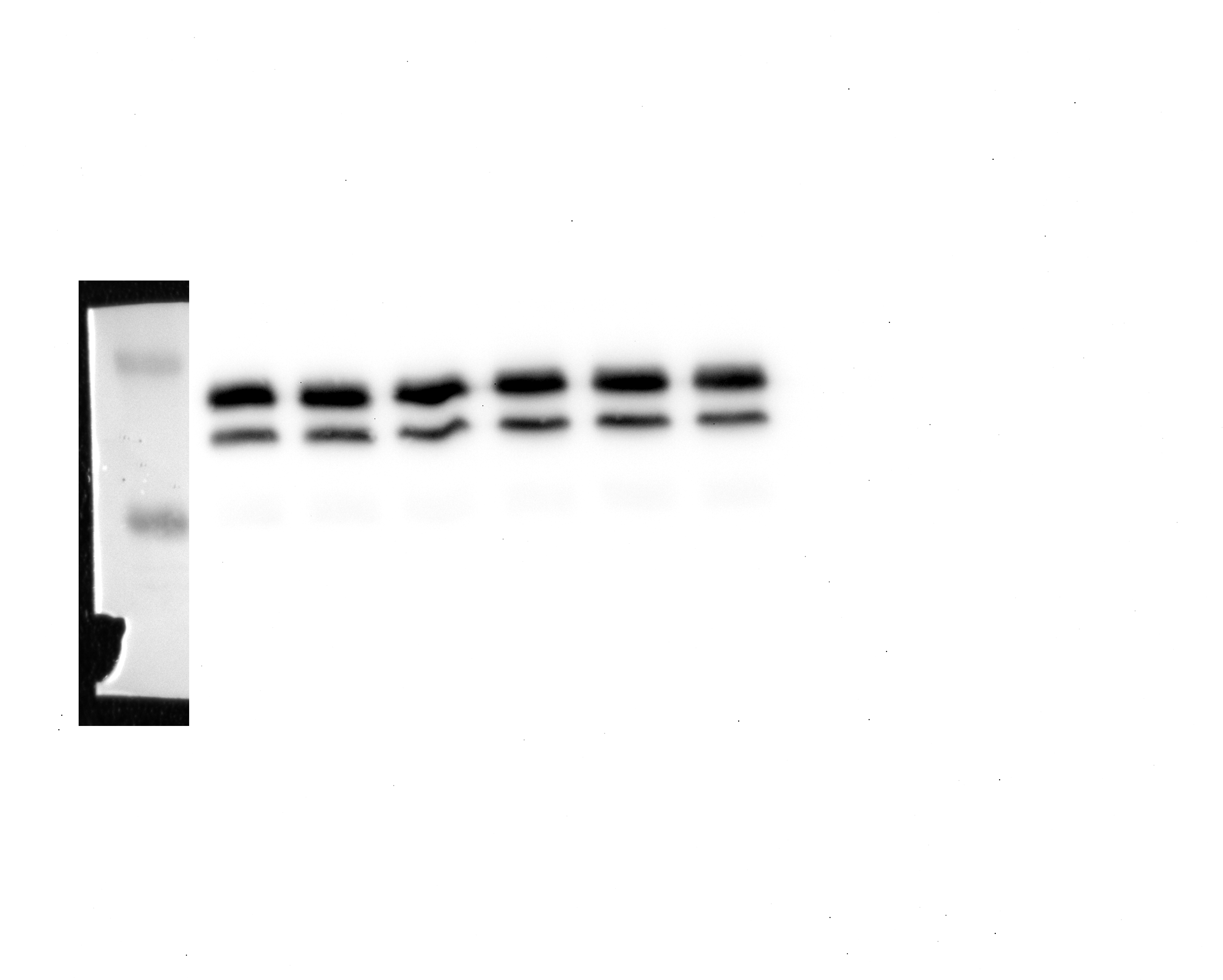

Supplement: Source data 1. [file elife-75523-data1.zip › Buscham Source Data Blots/Figure 2O Blot source data/Figure 2O PLP.tif]

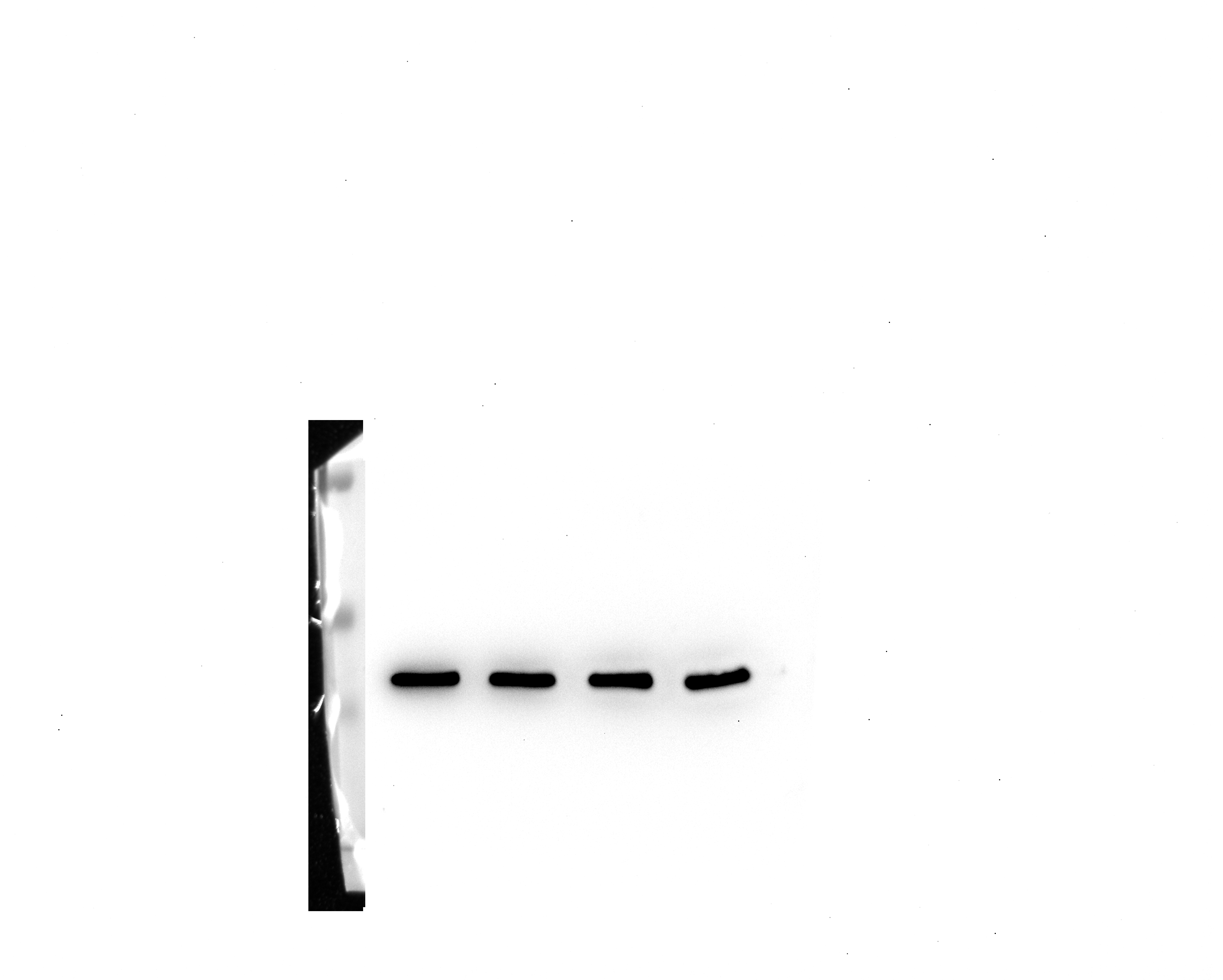

Supplement: Source data 1. [file elife-75523-data1.zip › Buscham Source Data Blots/Figure 6 Supplement 1A Blot source data/Figure 6 Supplement 1A CAII.tif]

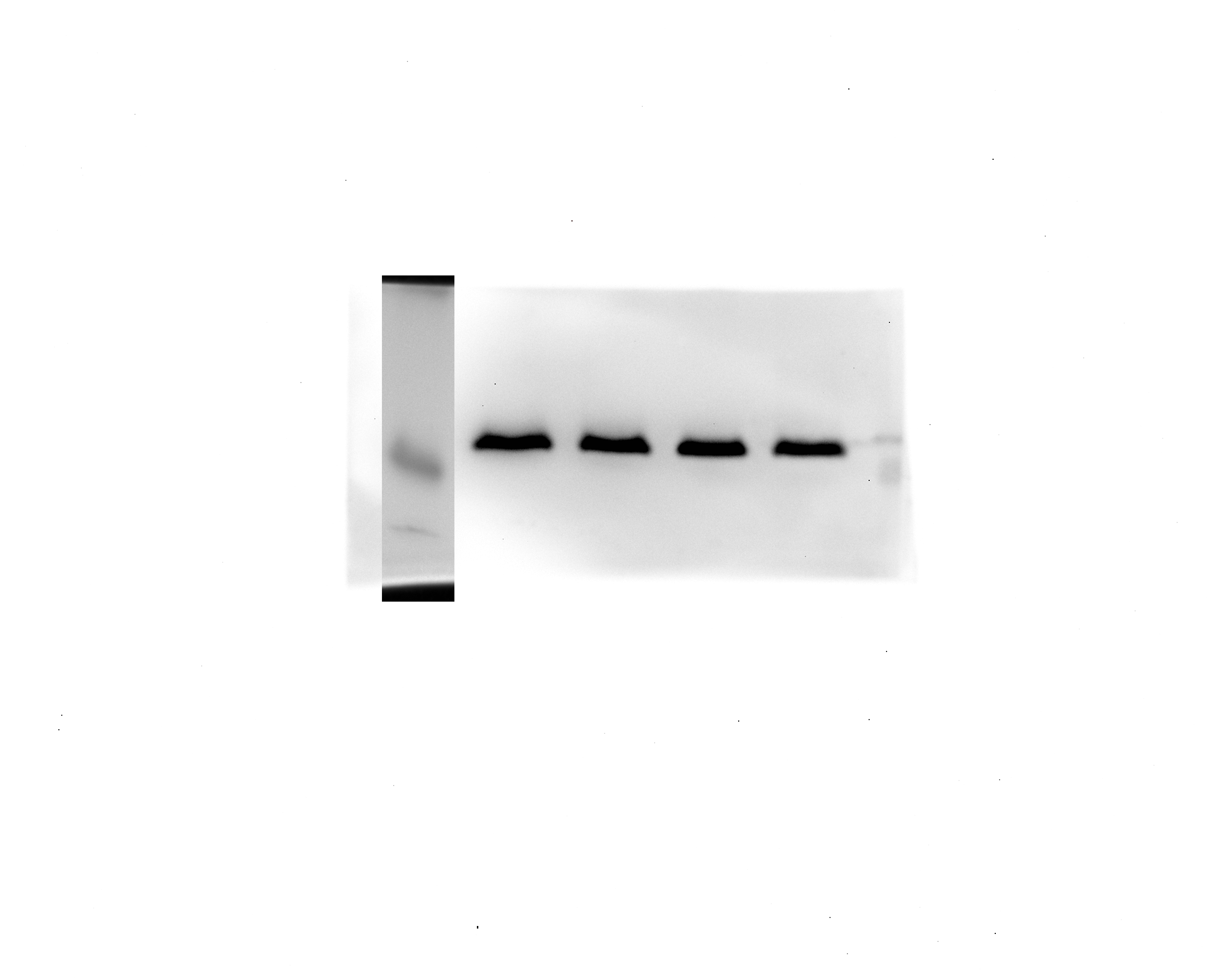

Supplement: Source data 1. [file elife-75523-data1.zip › Buscham Source Data Blots/Figure 6 Supplement 1A Blot source data/Figure 6 Supplement 1A CMTM5.tif]

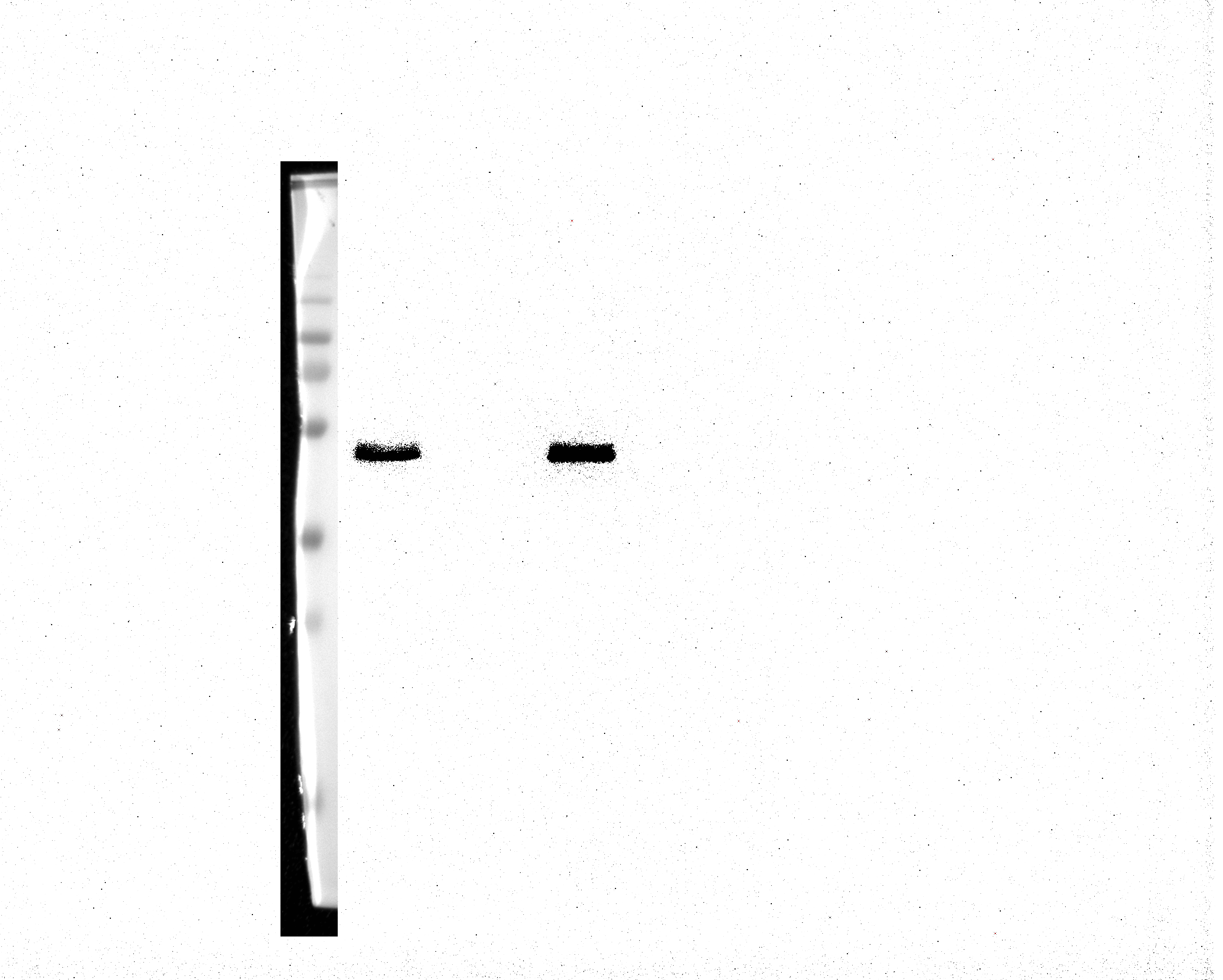

Supplement: Source data 1. [file elife-75523-data1.zip › Buscham Source Data Blots/Figure 6 Supplement 1A Blot source data/Figure 6 Supplement 1A CNP.tif]

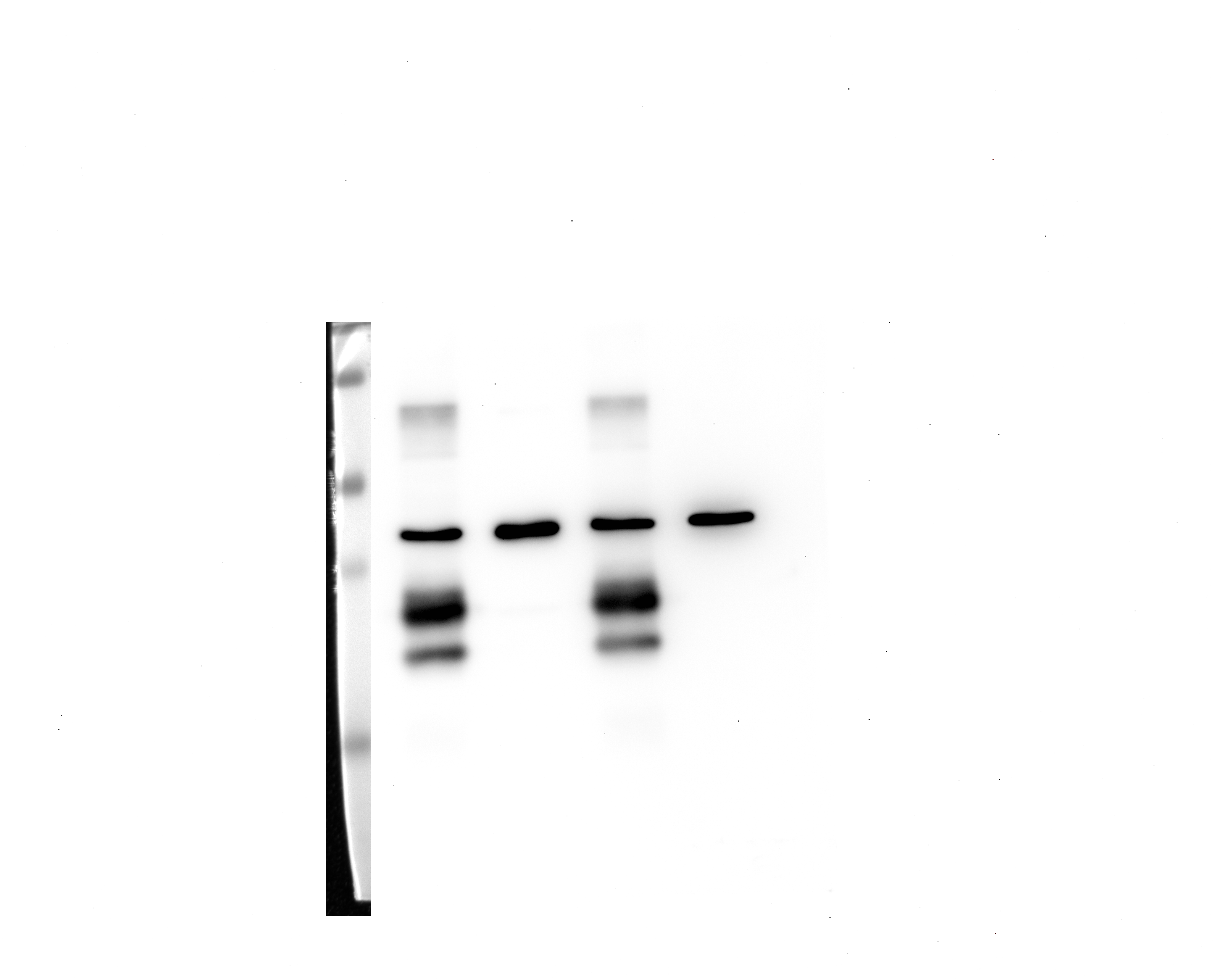

Supplement: Source data 1. [file elife-75523-data1.zip › Buscham Source Data Blots/Figure 6 Supplement 1B Blot source data/Figure 6 Supplement 1B CAII and PLP.tif]

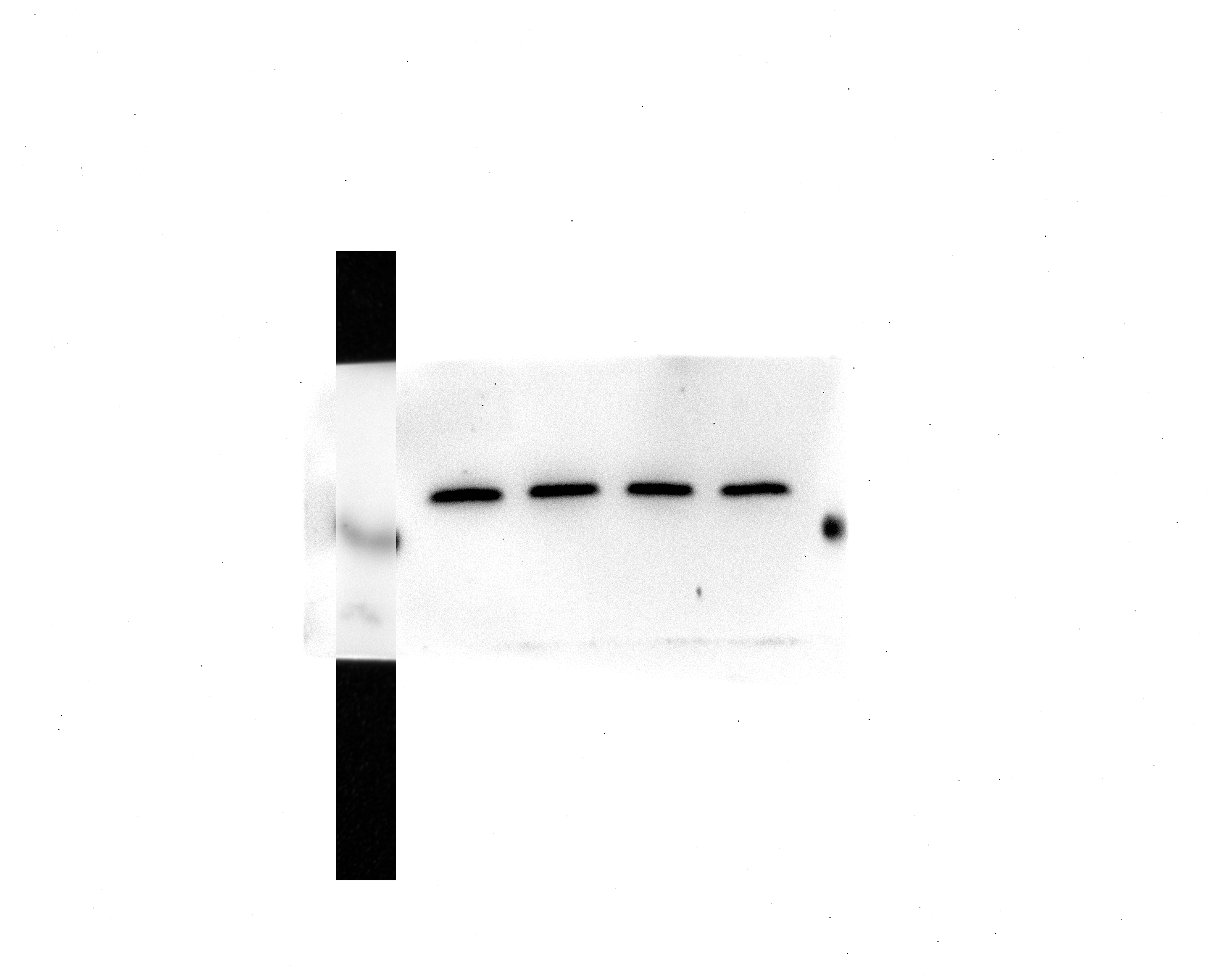

Supplement: Source data 1. [file elife-75523-data1.zip › Buscham Source Data Blots/Figure 6 Supplement 1B Blot source data/Figure 6 Supplement 1B CMTM5.tif]

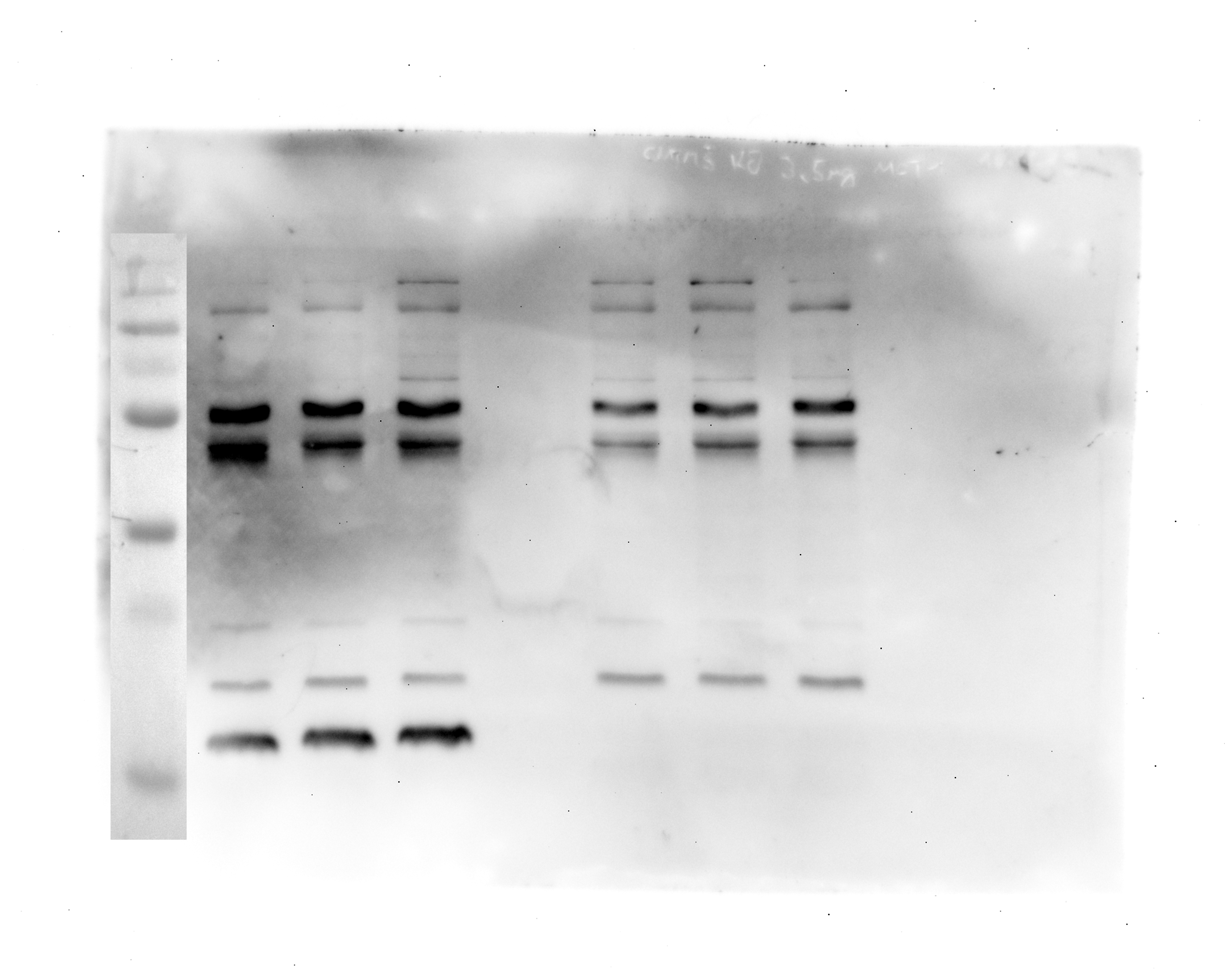

Supplement: Source data 1. [file elife-75523-data1.zip › Buscham Source Data Blots/Figure 6A Blot source data/Figure 6A CMTM5.tif]

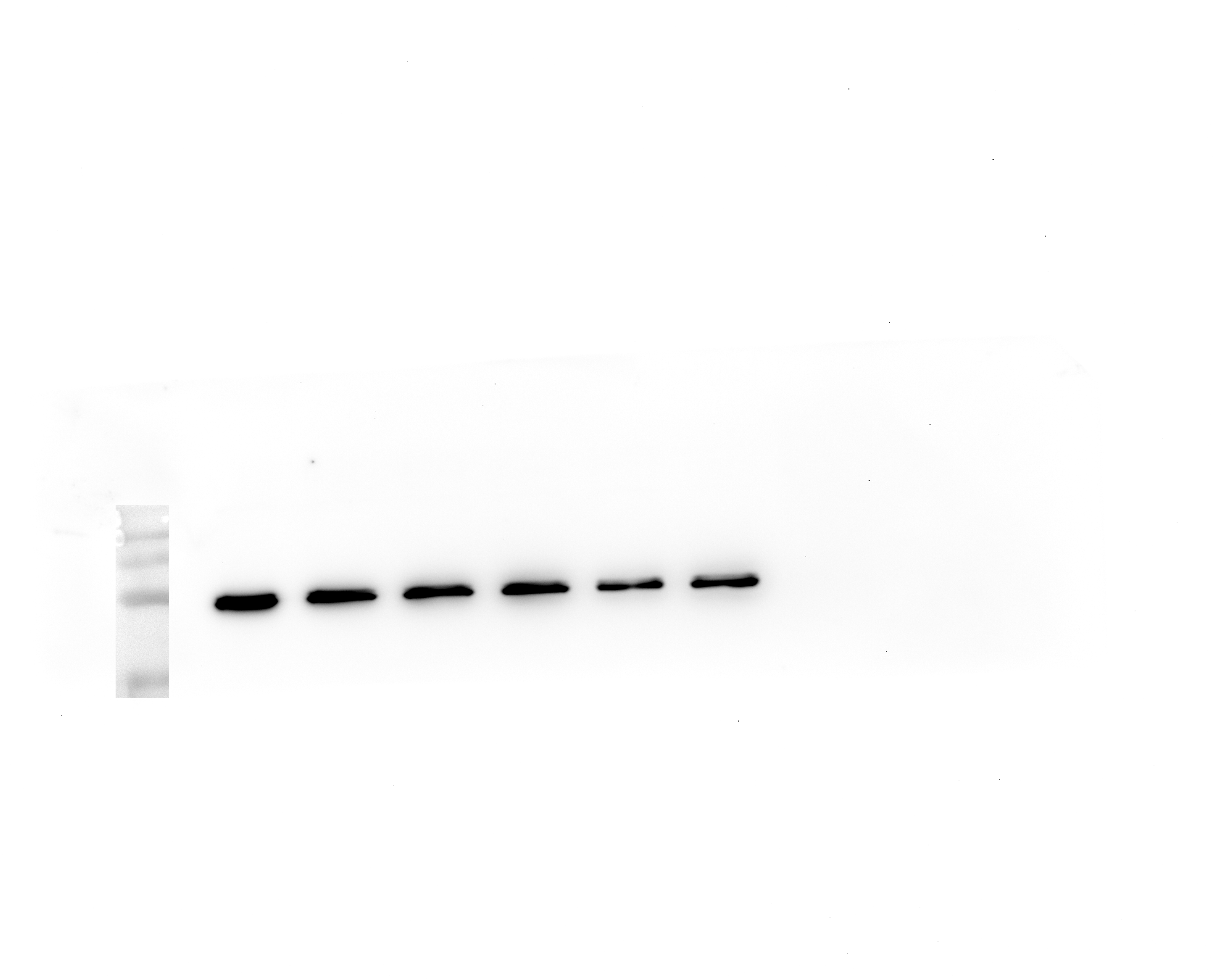

Supplement: Source data 1. [file elife-75523-data1.zip › Buscham Source Data Blots/Figure 6A Blot source data/Figure 6A CNP.tif]

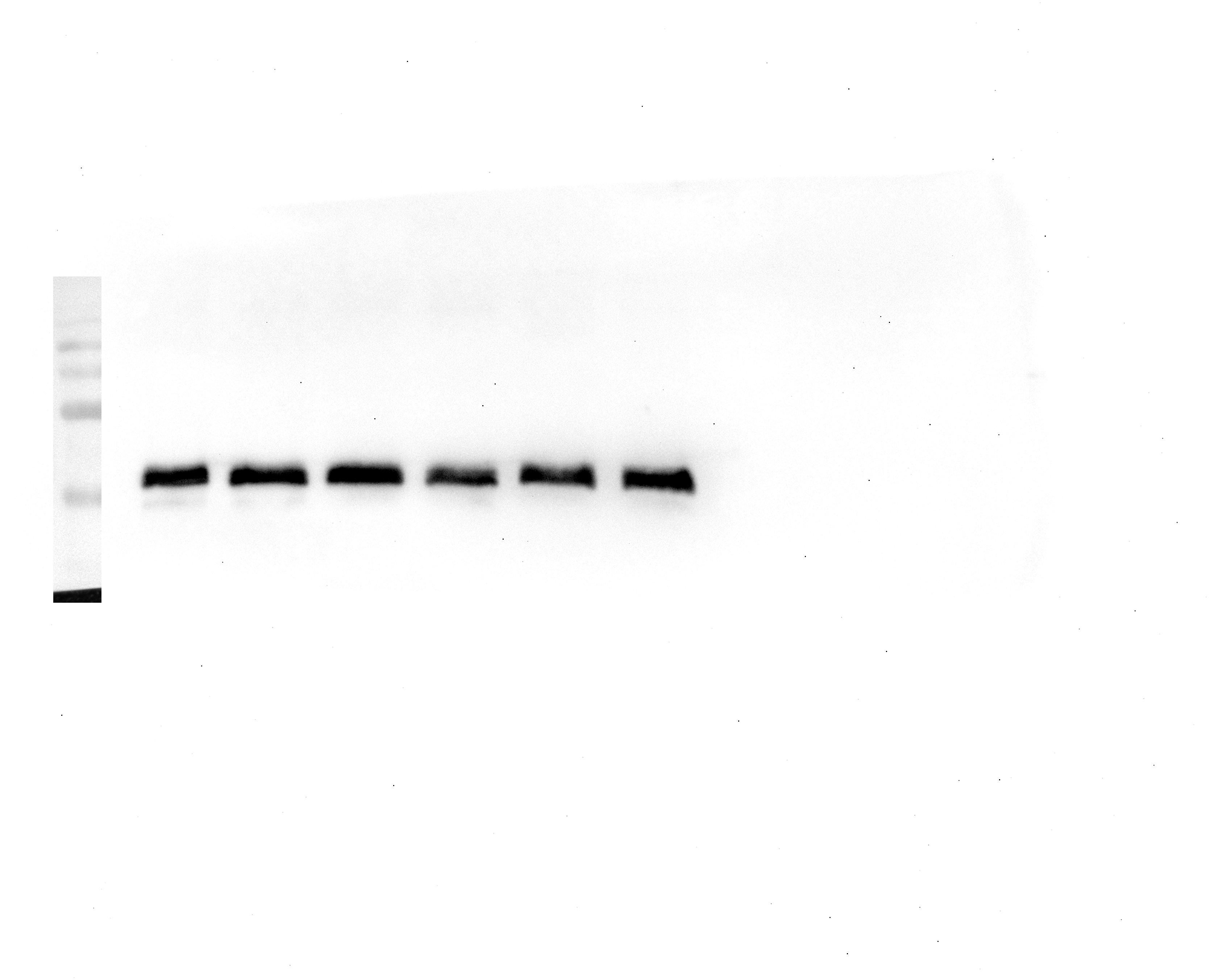

Supplement: Source data 1. [file elife-75523-data1.zip › Buscham Source Data Blots/Figure 6A Blot source data/Figure 6A SIRT2.tif]

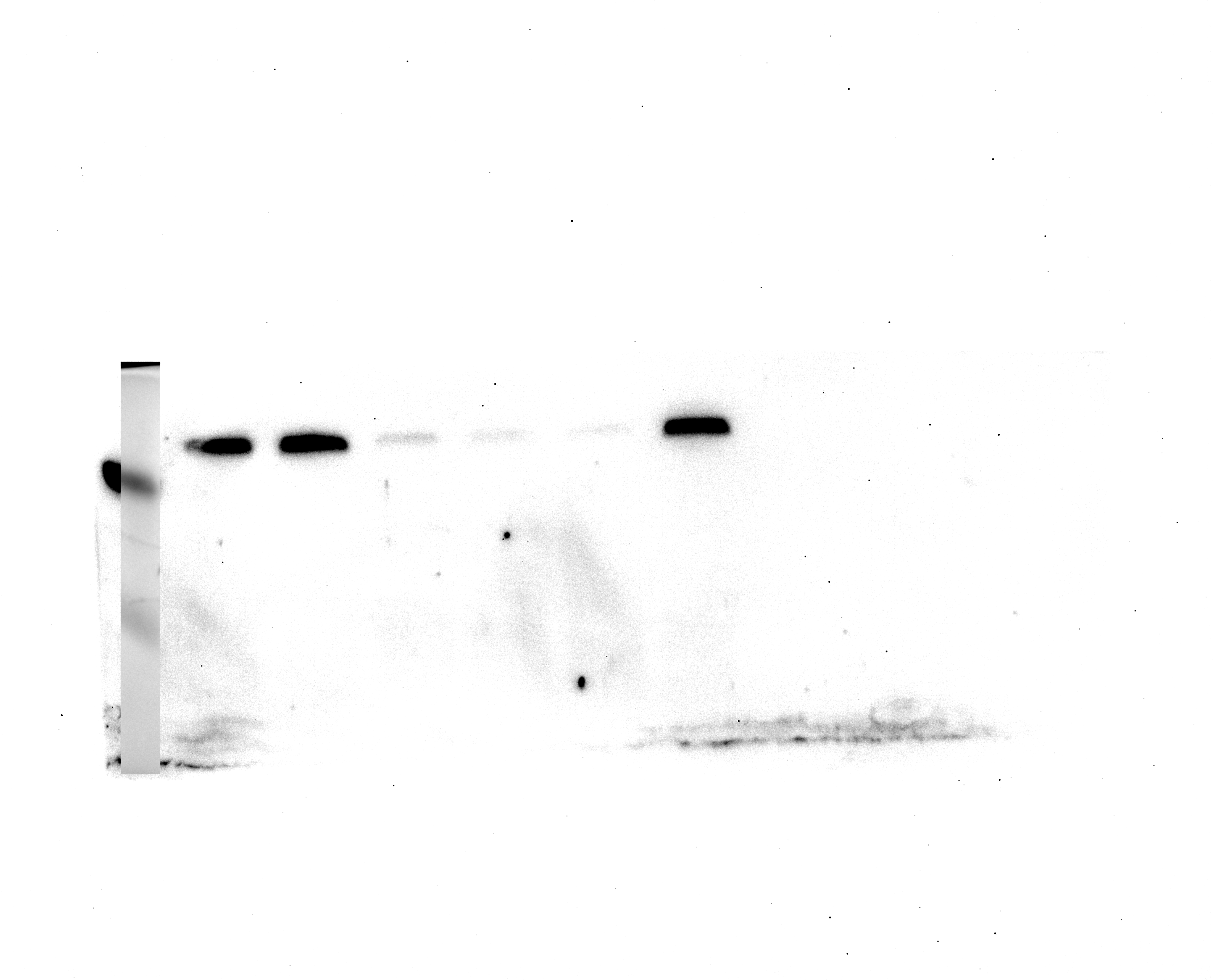

Supplement: Source data 1. [file elife-75523-data1.zip › Buscham Source Data Blots/Figure 6D Blot source data/Figure 6D CMTM5.tif]

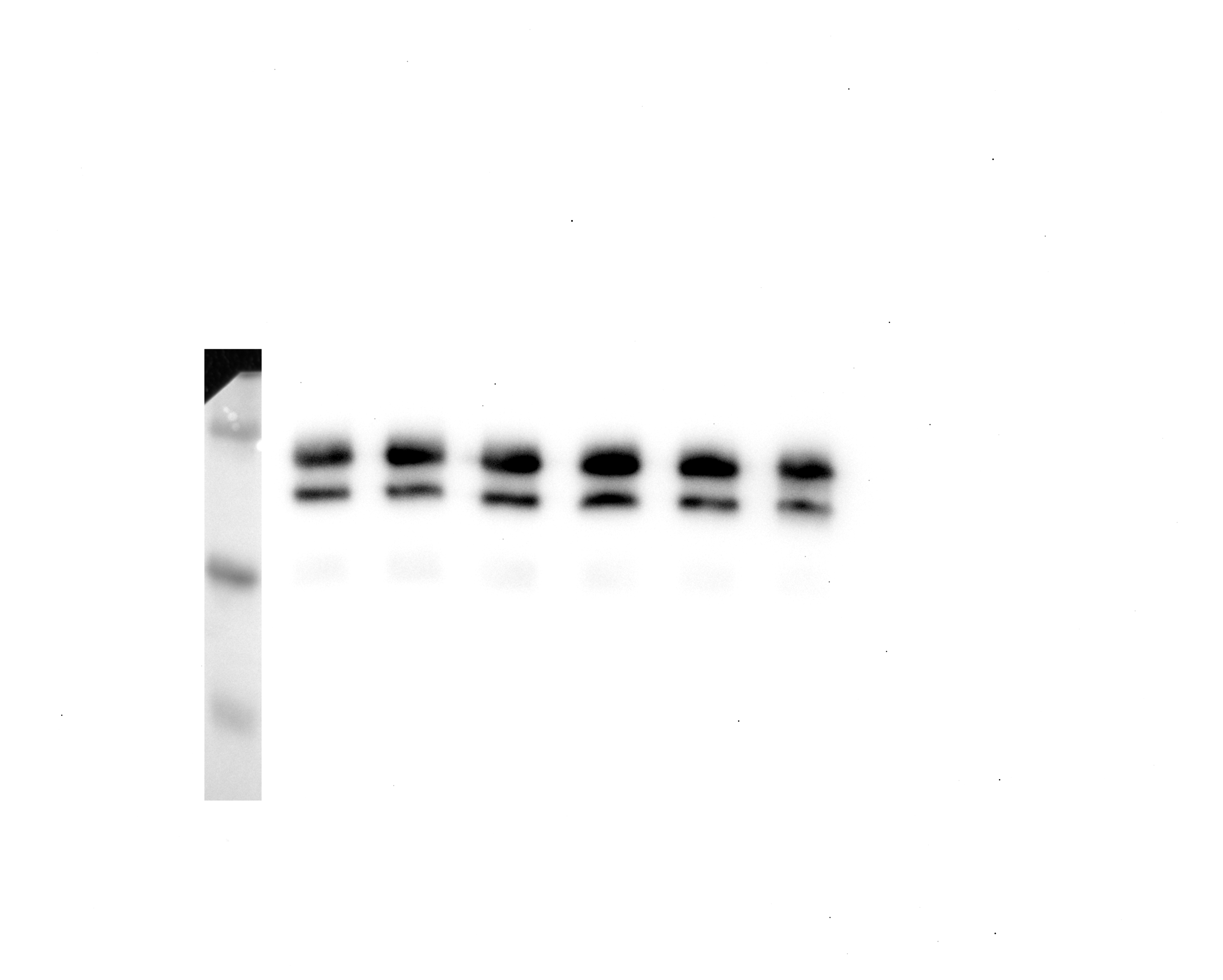

Supplement: Source data 1. [file elife-75523-data1.zip › Buscham Source Data Blots/Figure 6D Blot source data/Figure 6D PLP.tif]

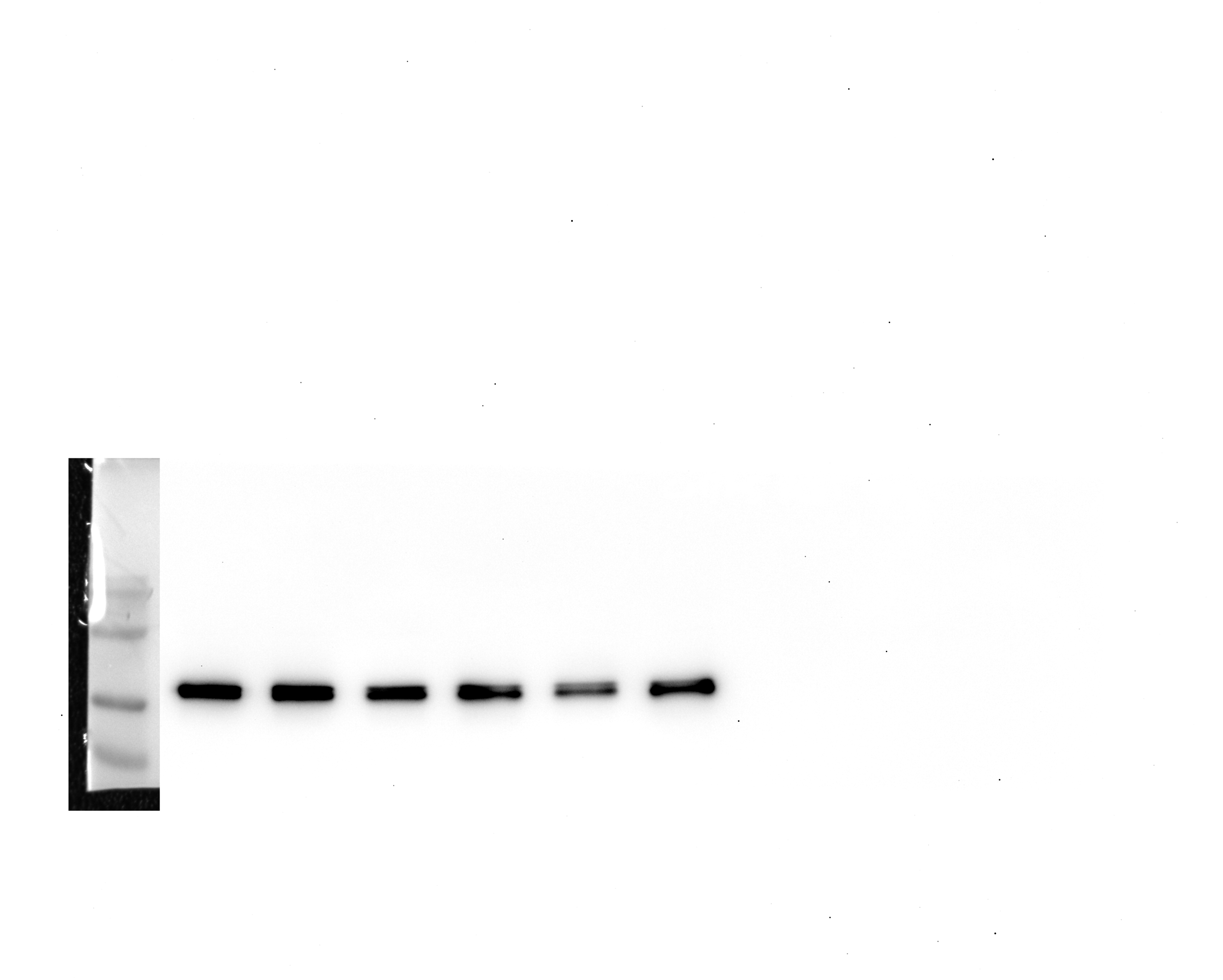

Supplement: Source data 1. [file elife-75523-data1.zip › Buscham Source Data Blots/Figure 6D Blot source data/Figure 6D SIRT2.tif]
